# Improved air-sea CO_2_ flux estimates from sailboat measurements

**DOI:** 10.1126/sciadv.adz1502

**Published:** 2026-01-09

**Authors:** Jacqueline Behncke, Tatiana Ilyina, Fatemeh Chegini, Peter Landschützer

**Affiliations:** ^1^ Max Planck Institute for Meteorology, Bundesstrasse 53, 20146 Hamburg, Germany.; ^2^ International Max Planck Research School on Earth System Modelling, Bundesstrasse 53, 20146 Hamburg, Germany.; ^3^ Universität Hamburg, Bundesstrasse 55, 20146 Hamburg, Germany.; ^4^ Helmholtz-Zentrum Hereon, Max-Planck-Straße 1, 21502 Geesthacht, Germany.; ^5^ Flanders Marine Institute (VLIZ), Jacobsenstraat 1, 8400 Ostend, Belgium.

## Abstract

Despite their importance in the climate system, remote ocean regions and their ability to absorb anthropogenic carbon dioxide (CO_2_) remain highly uncertain. To address this issue, citizen science initiatives, including sailboats, expand the observational network. Using observing system simulations and novel sailboat tracks, we demonstrate how integrating sailboat data improves estimates of ocean carbon uptake. While we underestimate the ocean carbon sink when mimicking real-world sampling, adding available sailboat data does not substantially improve reconstructions. Nevertheless, increased sampling reveals a stronger carbon sink, particularly between 40°S and 60°S. The improvement persists with hypothetical measurement uncertainties, but substantial differences arise depending on whether positive or negative biases are applied to the race track data. While we show that two additional circumnavigations already improve the ocean mean sink estimate, we further highlight the finding that the additional data remain insufficient to correct the overestimated CO_2_ sink trend, calling for continuation of the ongoing data collection.

## Teaser

Citizen science enables continuous sailboat sampling during global races, improving carbon uptake estimates in remote oceans.

## INTRODUCTION

The ocean plays a critical role in regulating Earth’s climate by acting as a substantial carbon sink that annually absorbs over a quarter of anthropogenically emitted carbon dioxide (CO_2_) from the atmosphere (*1*, *2*). However, climate change alters the carbon uptake capacity of the ocean (*1*, *3*), making the monitoring of the sea surface partial pressure of CO_2_ (pCO_2_) or the fugacity of CO_2_ (fCO_2_) indispensable in order to assess the impacts of climate change. Despite this need, a declining trend in observations in recent years and an imbalance in sampling efforts across the hemispheres persist (*4*, *5*). While the Ship of Opportunity (SOOP) program has successfully contributed to systematic sampling in the Northern Hemisphere (*6*), the Southern Ocean remains more irregularly sampled (*4*, *5*, *7*, *8*), leading to biases in the reconstruction of the air-sea CO_2_ flux (*9*–*12*). Specifically, insufficient sampling leads to a 31% overestimation of the Southern Ocean decadal variability (*9*) and a strong overestimation of decadal trends (*10*). Considering the key role of remote ocean basins such as the Southern Ocean in ocean carbon and heat uptake (*13*–*15*), and the moderate success of gap-filling methods in further improving fCO_2_ estimates (*9*, *10*, *16*), previously unexplored observational efforts have been undertaken to expand the observational network.

Sailboat races are emerging as a means to address observational gaps by providing a unique source of oceanographic data, particularly in remote ocean regions, as part of citizen science efforts (*17*, *18*). Despite their predominant occurrence in the North Atlantic, regular repeating circumnavigation races (Vendée Globe, every 4 years; The Ocean Race, every 3 to 4 years) also provide valuable data in the Southern Ocean—both regions prone to uncertainty in the air-sea CO_2_ flux (*19*, *20*) and drivers on multiple timescales (*21*). Hence, sailboats complement the observational network consisting so far of research ships, voluntary observing ships, drifting and moored buoys [e.g., (*22*)], gliders [e.g., (*23*–*26*)], biogeochemical floats [e.g., (*27*–*29*)], and Saildrones (*30*–*32*) and have been shown to significantly affect the air-sea CO_2_ flux estimate, particularly between 40°S and 60°S during austral summer (*17*). Previous research (*17*) has shown that even when considering potential measurement uncertainties within the range of ±5 μatm (1 atm = 101.325 kPa), the impact of new sailboat measurements on the air-sea CO_2_ flux is still detectable, unlike when considering a systematic measurement bias (*17*). What we still lack, however, is information on whether sailboat measurements actually improve the air-sea CO_2_ flux estimates, which has yet to be quantified. Here, we address this challenge.

Recent Observing System Simulation Experiments investigate the impact of different sampling strategies on carbon fluxes by using a model testbed as a benchmark to quantify the improvement (*10*, *27*, *30*, *33*–*36*). These investigations typically involve subsampling an fCO_2_ model testbed based on synthetic sampling schemes and reconstructing the air-sea CO_2_ flux with neural network gap-filling methods [e.g., (*37*)] to compare against the model truth. However, previous studies often focus on optimal, yet not operational, sampling scenarios rather than feasible real-world sampling schemes such as our repeating circumnavigations. Thus, although studies show promising improvements linked to optimized sampling (*10*, *38*), the implementation is often not feasible. The frequency of sailboat races allows us to quantify the extent to which realistic sampling by sailboats, particularly in the Southern Ocean, improves the estimate of the air-sea CO_2_ flux, providing a path forward in improved monitoring of the Southern Ocean CO_2_ uptake.

**Fig. 1.**
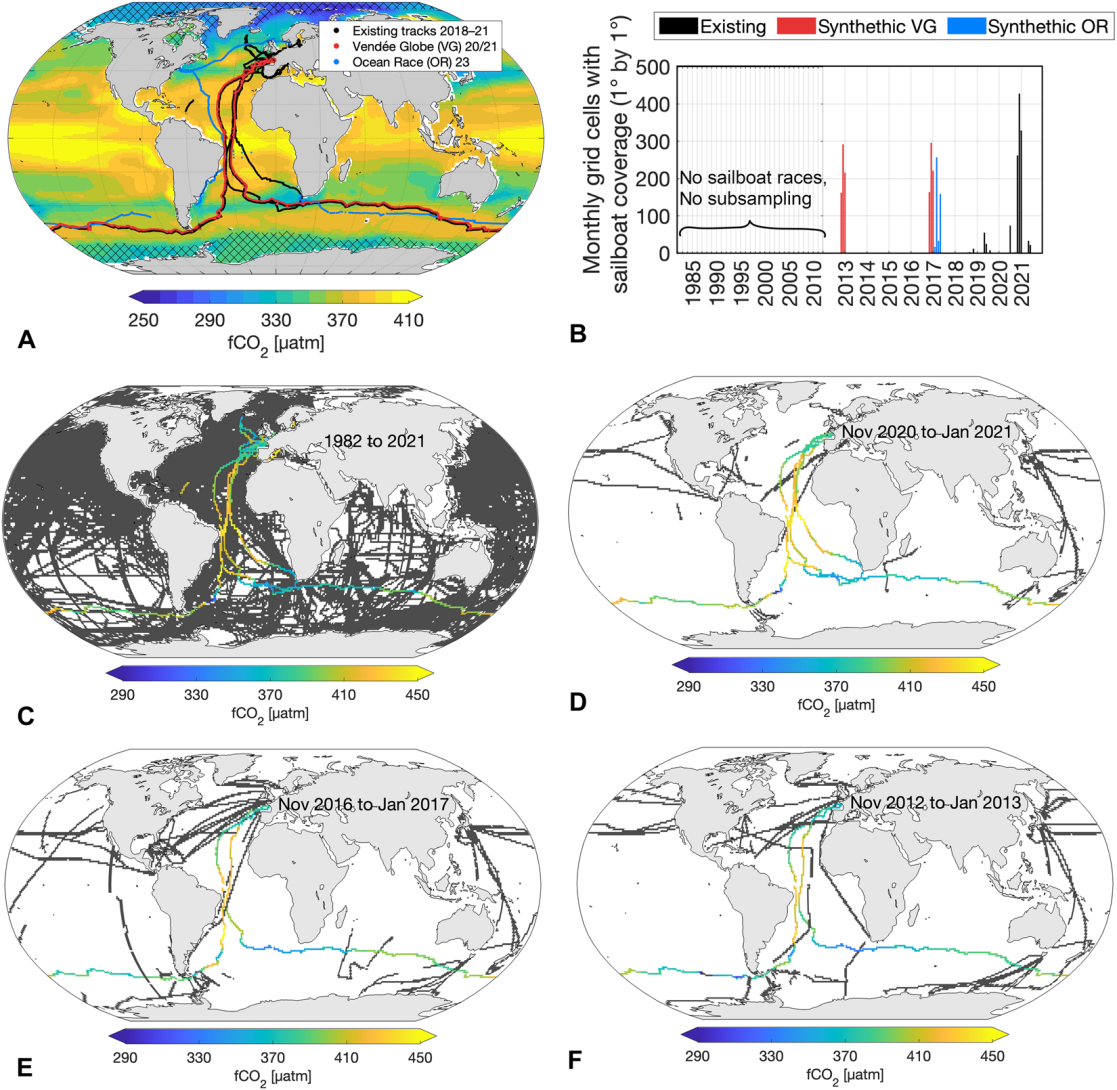
Sailboat sampling schemes. (**A**) Reconstructed fCO_2_ based on SOCAT sampling. The lines represent different sailboat tracks, which are used alongside the rest of SOCATv2022 tracks in the subsampling process [note: The Vendée Globe track (red) was plotted with a small offset from its original position to avoid overlap with the “existing sailboat” tracks, even though Vendée Globe 2020/21 is included in the “existing sailboat” tracks]. Hatched areas are regions with a climatological maximum sea-ice concentration greater than 50% and are excluded. (**B**) Sailboat data availability per month. Sampling scheme A “existing sailboat” = black data. Sampling scheme C “3 circumnav.” = black + red data. Sampling scheme D “2 diff. circumnav.” = black + blue data. Sampling scheme B “without sailboat” excludes all colored sailboat track data. (**C** to **F**) Tracks used to subsample the model during different time periods for the “3 circumnavigations” run. Gray lines indicate SOCAT tracks; colored lines represent subsampled sailboat fCO_2_ tracks.

Here, we conduct an observation system simulation by subsampling the global ocean biogeochemical model HAMburg Ocean Carbon Cycle (HAMOCC) coupled to the ocean general circulation model Max Planck Institute Ocean Model (MPIOM) that contributes to the Global Carbon Budget (*1*, *39*–*41*) mimicking the present-day observations [www.socat.info; (*4*)] of the sea surface fCO_2_ and apply the two-step neural network method self-organizing map–feed-forward neural network(SOM-FFN) (*37*, *42*) to reconstruct different air-sea CO_2_ flux estimates. We quantify potential improvements from our existing 161 days of sailboat data from several sailboats on the air-sea CO_2_ flux estimate as well as the potential effect of more data by subsampling data from previous years using realistic sailboat tracks (Fig. 1). Our analysis further explores the effect of random measurement uncertainties and systematic biases associated with the sailboat data. We investigate whether reconstructions continue to improve when additional, albeit biased, data are added, to assess whether the increase in quantity compensates for the lack of quality. Here, we show that continuous and long-term observing improves the mean air-sea CO_2_ flux estimate; however, our analysis reveals that the reconstructed trend from the neural network method remains overestimated even after adding “3 circumnavigation” races, highlighting the need for multidecadal observing strategies.

## RESULTS

In this study, we show how incorporating different synthetic sailboat data improves the reconstructions of a known-truth model field in an observing system simulation experiment. We take the full hindcast model fCO_2_ field from MPIOM-HAMOCC and subsample it at times and locations where (based on a 1°-by-1° grid location) historical and synthetic observations exist. Figure 1 presents the different pseudo-observation tracks used in this study to subsample the model field.

Using the SOM-FFN method, i.e., a two-step neural network method [see (*37*) for details], we then gap-fill these subsampled data to reconstruct the complete model fCO_2_ field. Figure 1A presents the reconstructed global fCO_2_ based on subsampled model data mimicking the real-world observations from Surface Ocean CO_2_ Atlas (SOCAT) (“existing sailboat”). After deriving the air-sea CO_2_ flux estimates from the subsampled and gap-filled fCO_2_ field, our analysis proceeds in three steps: (i) assessing the neural network’s performance with present-day sampling against the model truth; (ii) quantifying improvements in air-sea CO_2_ flux estimates from adding different sailboat data tracks; and (iii) assessing the impact of measurement uncertainties and biases.

### Performance of neural-network reconstruction

We evaluated the neural network method’s performance in reconstructing fCO_2_ (based on available present-day observation tracks from SOCATv2022 “existing sailboat”) by comparing the fCO_2_ model truth with the “existing sailboat” fCO_2_ estimate. By comparing fCO_2_ data distributions, we observe a relatively strong agreement (fig. S1). The neural network successfully reconstructed fCO_2_ values close to the model truth, with the exception of the polar north (fig. S1), i.e., a region that has been identified as erroneous in previous studies (*39*, *42*) and is thus not further considered here (see Materials and Methods). Probability density functions for the original model fCO_2_ and the model-subsampled neural-network-reconstructed fCO_2_ show dissimilarities with Bhattacharyya distances (BDs) ranging from 0.00 to 0.14. The BD (*43*) measures the similarity between two probability distributions, with lower values indicating greater similarity. In this context, the distances ranging from 0.00 to 0.14 suggest a high degree of similarity between the distribution of the model fCO_2_ and reconstructed fCO_2_.

The fCO_2_ distributions in the tropics show the highest agreement with relatively low BDs, suggesting a robust representation of the observed patterns, and almost identical means (0.23 to 1.11 μatm difference) between model and reconstruction followed by the fCO_2_ distributions in the middle latitudes (fig. S1). In the Southern polar region, dissimilarities are more pronounced, featuring BD between 0.04 and 0.05 and higher offsets between mean fCO_2_ values ranging from 0.92 to 3.78 μatm (fig. S1).

**Fig. 2.**
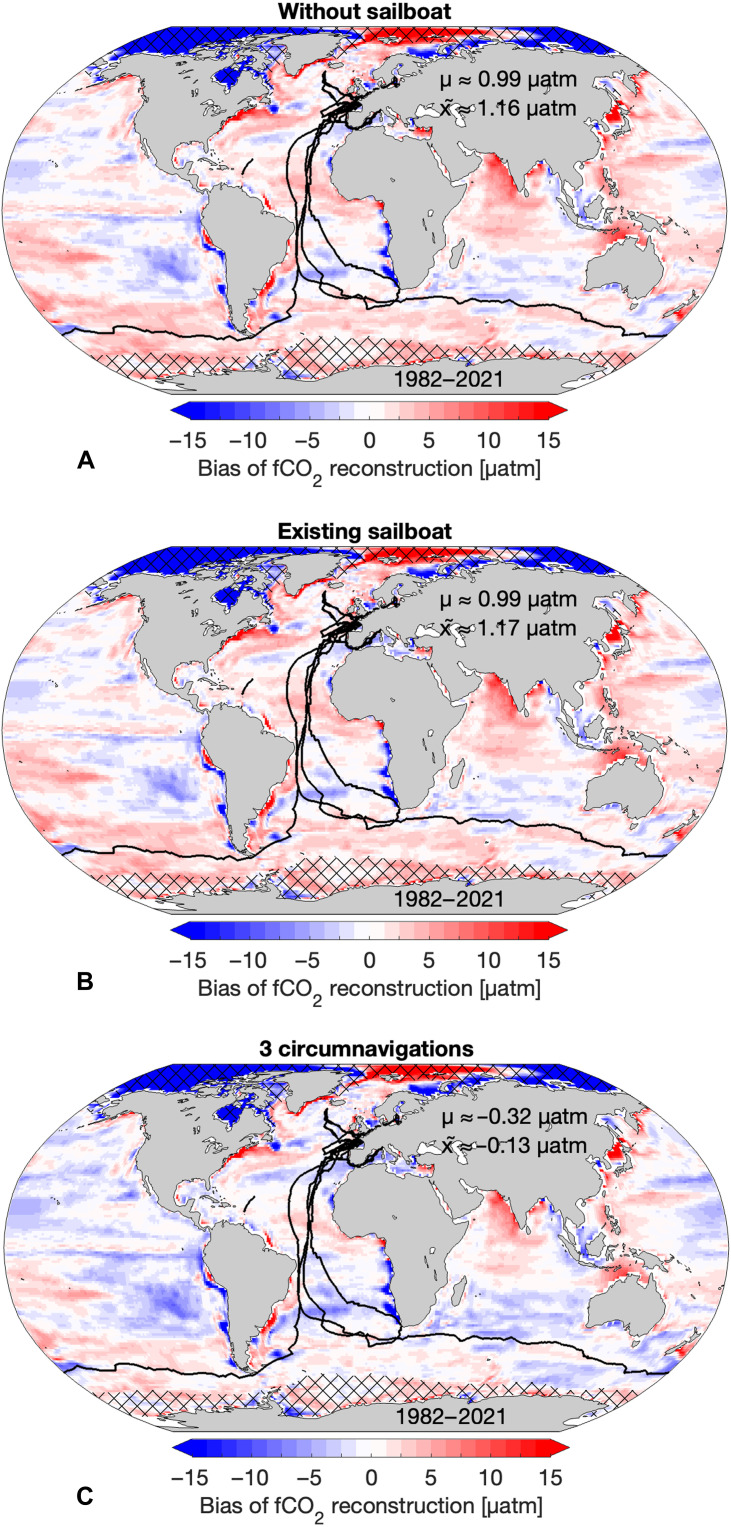
Spatial fCO_2_ bias and improvement patterns resulting from the integration of different sampling schemes. (**A** to **C**) Mean bias of subsampled and reconstructed fCO_2_ averaged over 1982 until 2021 based on data subsampled after SOCAT (A) “without sailboat” track data B (B) including “existing sailboat” tracks A (C) including existing sailboat tracks and 2 additional circumnavigations (C or “3 circumnav.”). Black lines represent sailboat tracks. Hatched areas are regions with a climatological maximum sea-ice concentration greater than 50% and are excluded. μ represents the mean, while x̄ represents the median.

Both over- and underestimation of fCO_2_ occur along the coastal ocean (Fig. 2), a region that is highly variable (*44*, *45*). We find that the neural network in combination with the present-day sampling overestimates fCO_2_ in the undersampled Southern Ocean by around 2 to 3 μatm (Fig. 2B and fig. S2). A similar overestimation of fCO_2_ was found in (*30*) using a different reconstruction method and a large ensemble testbed of Earth System Models. In contrast, while similar in magnitude to our results, the sign of the mismatch is opposite to a study conducted in (*10*) using a different single hindcast model, illustrating the limitations of such an analysis to a single model. However, here, we are interested in potential improvements in space and time from adding measurement compared to the baseline. In addition, we do not test the effect of different gas transfer schemes, as our study solely focuses on the improvement in the fCO_2_ from increased sailboat sampling. We find that additional sailboat data from “3 circumnavigations” already reduces the fCO_2_ in the Southern Ocean, hence improving the reconstruction (fig. S3).

Behncke *et al.* (*17*) quantified the detectable change caused by the addition of existing sailboat data based on real-world observations. To validate this and determine the similarities between observation and model data, we applied the same method to our subsampled model data and compared reconstructions based on subsampled model data with those based on observations. The neural network demonstrated consistent but smaller signal detection in subsampled model data, mirroring observations closely, thus underscoring the method’s realistic performance on both model and observation data (fig. S4).

The effect of the sailboat sampling scheme on the model reconstruction is more subtle, complicating detection as the relationships between driver variables and fCO_2_ are more consistent in the model setup, compared to the real world. Measured fCO_2_ is subject to variability and uncertainties, such as measurement uncertainties and temporal averaging when producing gridded fields; fCO_2_ measurements taken at one point in time are averaged per month, reducing accuracy. In addition, the effect in observations may be more pronounced because effects of hypothetical biogeochemical and physical processes and their variability that could be missing or unresolved in models might be captured in the real world (*39*, *46*–*48*).

### Effect of sailboat sampling on fCO_2_ estimates

Adding data from the existing sailboat tracks does not substantially reduce the fCO_2_ bias when averaged over the full time period 1982–2021, probably due to their small number and their late addition to the SOCAT database (Fig. 2, A and B). The global mean fCO_2_ is overestimated by 0.99 μatm regardless of the inclusion of sailboat data and particularly in the Southern Ocean by around 3 μatm (Fig. 2, A and B, and fig. S2). Similarly, the median bias is 1.17 μatm with sailboat data and 1.16 μatm without it. The spatial bias pattern stays nearly identical (Fig. 2A). In contrast, adding sailboat fCO_2_ data from “3 circumnavigation” tracks substantially reduces biases mainly in the North Atlantic and in the Southern Ocean, mirroring the data addition in these regions (Fig. 2, A to C). The fCO_2_ estimate improves with the addition of “3 circumnavigations” particularly between 40°S and 60°S (Fig. 2 and fig. S2), similar to findings in (*17*). Due to the neural network’s capacity to extrapolate over time and space, fCO_2_ estimates in other regions, e.g., in the Indian Ocean and West Pacific, where we do not add new data, improve as well (Fig. 2, B and C). However, we also observe compensating effects, indicating a trade-off in other regions, e.g., the South Pacific (Fig. 2, B and C). While the neural network learns new features and minimizes errors, it also adopts inaccurate representations of processes due to overfitting tendencies, resulting in increased biases in, e.g., parts of the East Pacific, where the fCO_2_ is underestimated. Globally, however, the median bias decreases to near zero, when adding data from “3 circumnavigation” tracks (Fig. 2).

### Effect of sailboat sampling on air-sea CO_2_ flux estimates

**Fig. 3.**
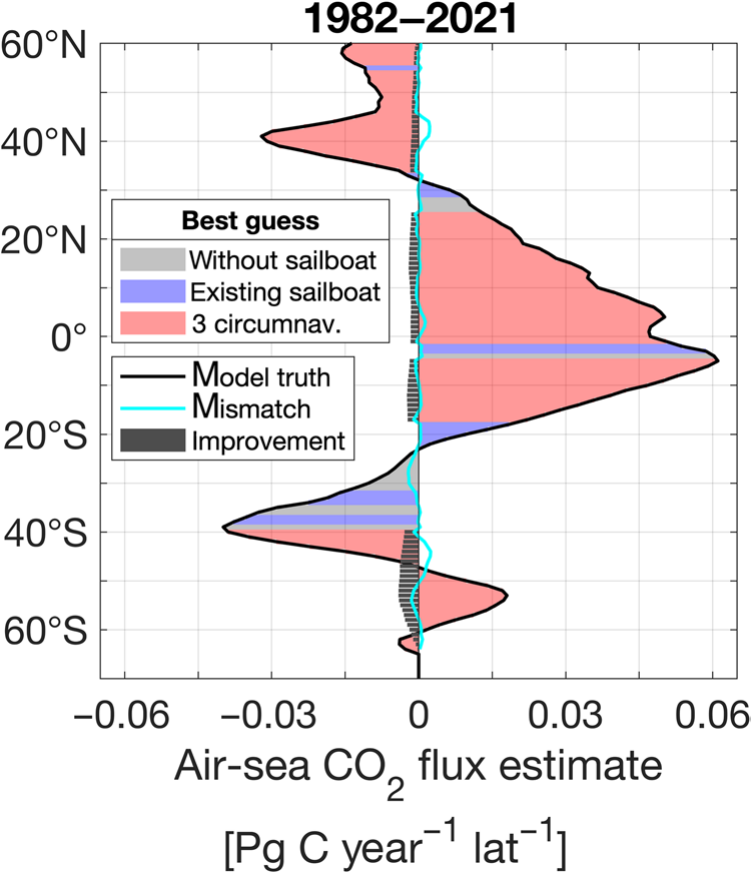
Latitudinal air-sea CO_2_ flux estimates for the period from 1982 to 2021. The black line represents the model truth. The area under the black curve is shaded to depict the “best guess” estimate closest to the model truth, with the color corresponding to one of three scenarios: A (“existing sailboat”), B (“without sailboat”), or C (“3 circumnav.”). The cyan line highlights the persistent mismatch between the “best guess” estimate and the model truth. Gray bars illustrate the improvement in estimates from scenario B (“without sailboat”) to the “best guess.” For example, bars pointing in the negative direction indicate that the “best guess” reduced the estimate by that amount. A cyan line pointing to the left indicates that the estimate is still too negative, while a line pointing to the right indicates that the estimate is still too positive.

Using these fCO_2_ reconstructions and model wind data, we compute the air-sea CO_2_ flux estimates by applying a bulk gas transfer formulation (fig. S5) (*37*, *49*). Figure 3 shows the latitudinal air-sea CO_2_ flux estimates and the best guess closest to the model truth with the direction of improvement. Mirroring the spatial improvement pattern seen in the fCO_2_ estimate (Fig. 2), the air-sea CO_2_ flux estimate improves the most with the addition of “3 circumnavigations,” particularly between 40°S and 60°S (Fig. 3), similar to findings in (*17*). This results in a more negative air-sea CO_2_ flux, indicating an enhanced global ocean carbon sink (Fig. 3). In contrast, we observe no improvement of the reconstruction between 20°S and 40°S (Fig. 3), where the model truth sink is weaker than estimated with more sailboat data.

**Fig. 4.**
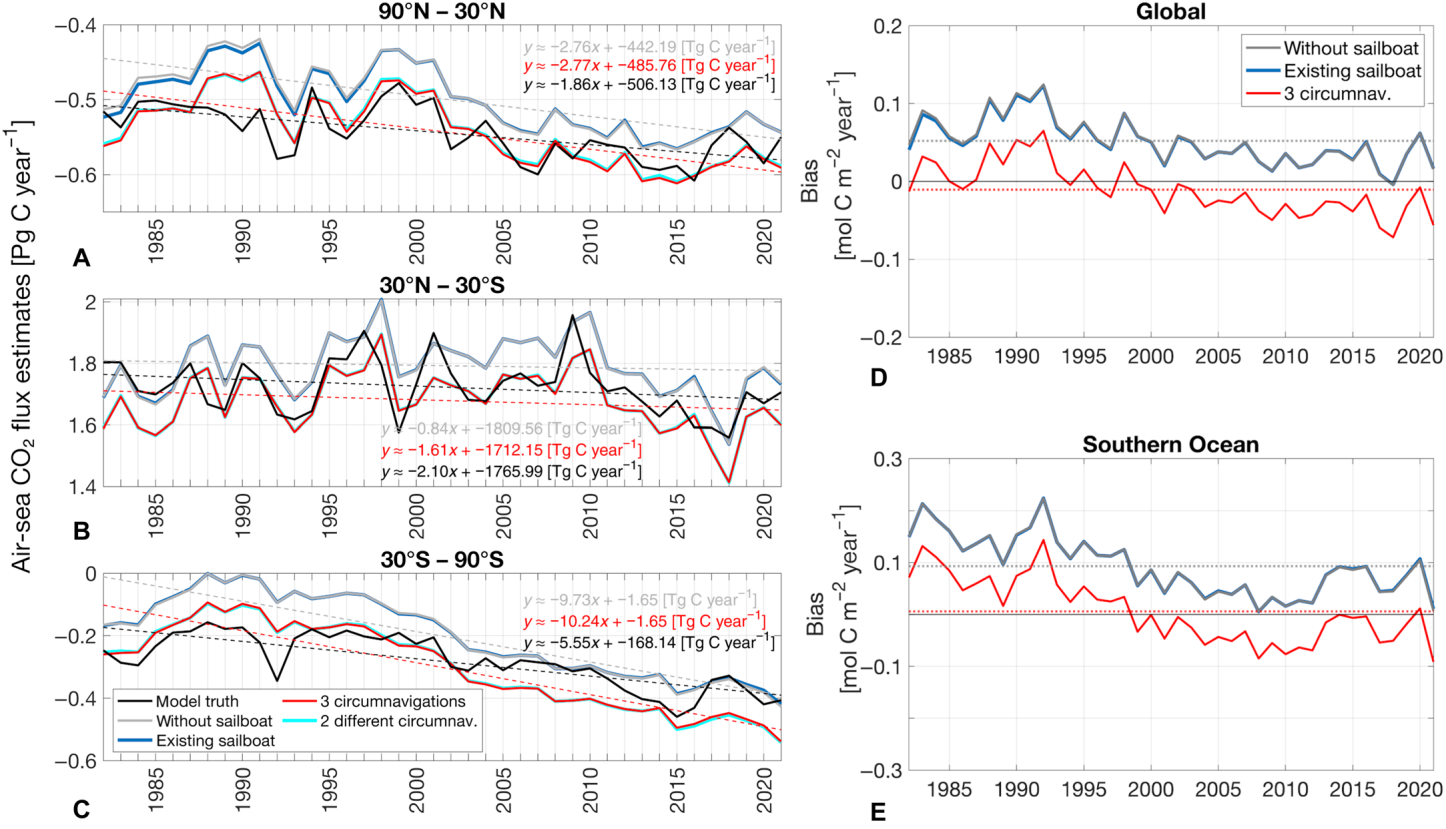
The impact of adding sailboat data on air-sea CO_2_ flux time series. (**A** to **C**) Air-sea CO_2_ flux estimates with and without different sailboat tracks in different regions. Dashed lines show the long-term trend. (**D** and **E**) Time series of bias (global and in Southern Ocean) in reconstructed air-sea CO_2_ flux density. Dotted lines represent the long-term mean bias. Note that the gray line, indicating the bias of the estimate based on “without sailboat,” and the blue line, indicating the bias of the estimate based on “existing sailboat,” are in close proximity to each other. Regions with a climatological maximum sea-ice concentration greater than 50% are excluded.

In addition to spatial patterns, we evaluate the influence of added sailboat data on the temporal trajectory of the air-sea CO_2_ flux (Fig. 4). Similar to the estimated fCO_2_, the air-sea CO_2_ flux does not show substantial improvement with the addition of existing sailboat tracks (see proximity of gray and blue lines in Fig. 4, A to E). However, the global mean air-sea CO_2_ flux density bias slightly decreases from 0.06 to −0.02 mol C m^−2^ year^−1^, and in the Southern Ocean, it improved from 0.10 to 0.00 mol C m^−2^ year^−1^ when data from two additional circumnavigation tracks (“3 circumnavigations”) were included (Fig. 4, D and E). Without these data, we observe that the annual air-sea CO_2_ flux is almost consistently too positive, indicating excessive outgassing and/or insufficient uptake (see gray lines in Fig. 4). However, when data from “3 circumnavigation” tracks are added, the air-sea CO_2_ flux estimate decreases by ~0.1 mol C m^−2^ year^−1^. This adjustment brings the estimate closer to the model truth (Fig. 4) by reducing the excessive outgassing in the tropics (Fig. 4B) and increasing the insufficient uptake north and south of 30° before 2000 (Fig. 4, A and C). It also results in an exaggeration of the total carbon sink after 2000 on a global scale (Fig. 4, A to C and D) and in the Southern Ocean (Fig. 4, C and E) as a trade-off.

The inclusion of additional sailboat data not only affects the air-sea CO_2_ flux where the data have been added, but the neural network extrapolates in the past, affecting the air-sea CO_2_ reconstructions consistently over time. Even though no new data were added prior to 2012/2013, the data addition in the later years influenced the pre-2000 air-sea CO_2_ flux estimate (Fig. 4, A to E), similar to results shown in (*30*), probably due to the sparse data during that period, as less than 1% of the monthly 1°-by-1° grid cells contain measurements (*5*). In contrast to the findings in (*17*, *27*), where the effect of additional data on the air-sea CO_2_ flux was primarily detected in the most recent years, attributed to the use of atmospheric CO_2_ as a predictor with trend behavior, our study demonstrates improvement over the entire time series. This more generalized impact, similar to (*30*), is mainly attributed to the increased data volume spanning multiple years as well as methodological differences such as the different nature of data.

The larger dataset, covering, e.g., a decade starting in the “3 circumnavigation” run, offers broader temporal coverage. This allows the model to learn generalized patterns and make informed predictions further back in time, enhancing its ability to extrapolate beyond the immediate training period. In addition, the use of model data, instead of observations, provides consistent relationships between fCO_2_ and predictors that enable the neural network to learn and generalize patterns more effectively.

In the Southern Ocean, the air-sea CO_2_ flux density is improved before 2000 and in some of the better observed recent years when adding “3 circumnavigations”; however, it notably worsens between 2005 and 2012 as a trade-off for improving the full time series (Fig. 4, C and E and fig. S6, C and D). Globally, the estimate is most improved before 2000, but it also continues to show improvement afterward (Fig. 4, A to D, and fig. S6, A and B).

Our findings further reveal a remarkable behavior: While we observe a shift in the mean due to the addition of the measurements, the shape of the time series and, consequently, the air-sea CO_2_ flux anomalies and trend (represented by dashed lines in Fig. 4, A to C) remains unchanged and continues to be overestimated compared to the model truth trend, particularly in the Southern Ocean, even with the addition of data from two additional circumnavigation tracks (Fig. 4).

Adding data from one additional but different circumnavigation track, such as from The Ocean Race 2023 instead of the Vendée Globe 2020/21 (Fig. 1), to the existing sailboat data improves the air-sea CO_2_ flux estimates as effectively as adding data from two identical Vendée Globe 2020/21 tracks (see cyan and red lines in Fig. 4).

### The effect of measurement uncertainties and biases on the air-sea CO_2_ flux estimate

We explore the effect of two types of measurement errors applied to the subsampled sailboat fCO_2_ data on the resulting air-sea CO_2_ reconstructions: random measurement uncertainties, representing zero-mean fluctuations in the measurements, and systematic biases, representing constant offsets. Random uncertainties are assumed to be normally distributed around zero, while biases introduce a fixed, consistent deviation from the true value.

**Fig. 5.**
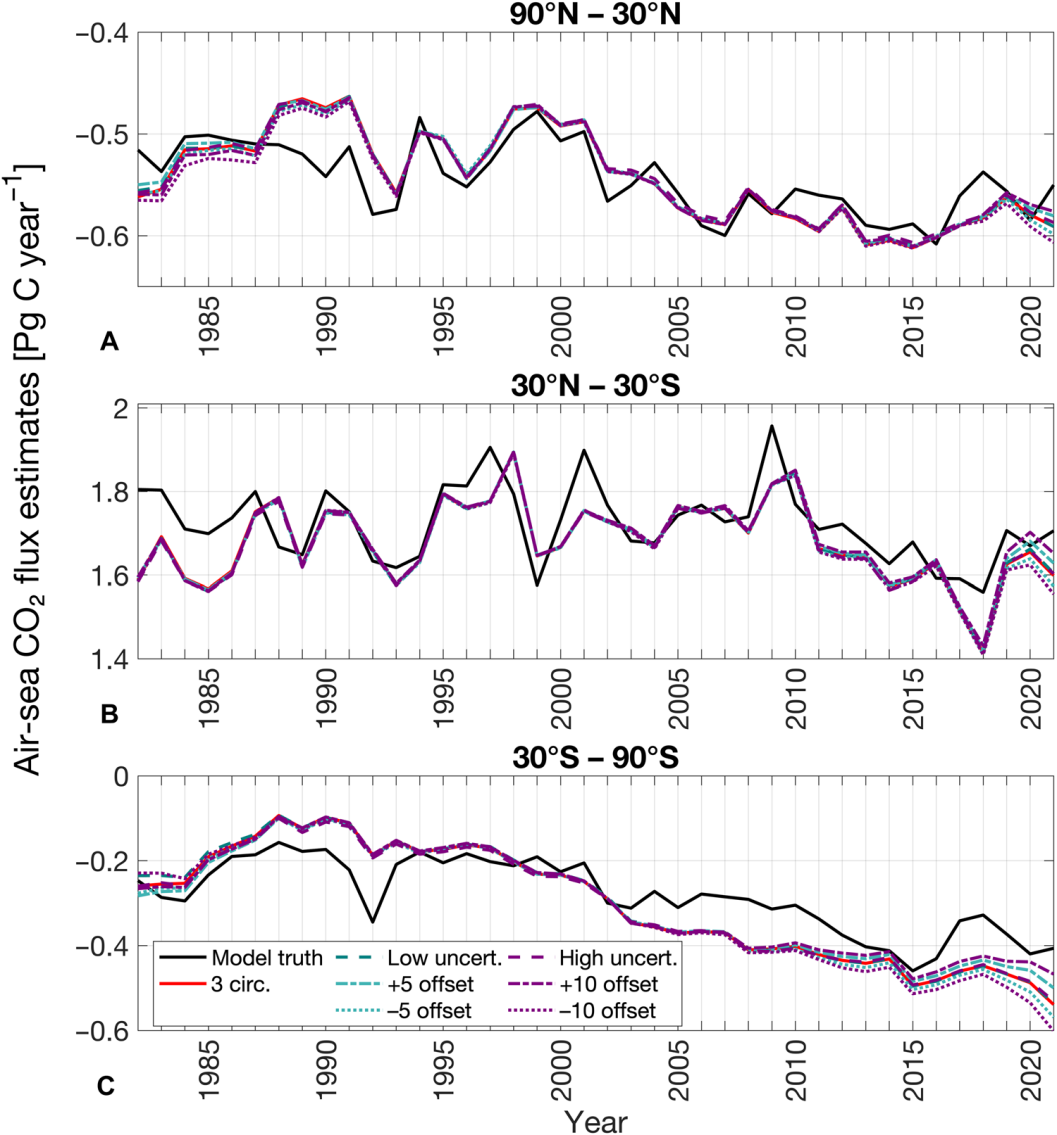
The effect of measurement uncertainties and biases on the air-sea CO_2_ flux estimate. (**A** to **C**) Air-sea CO_2_ flux estimates based on the model truth (solid black lines), the “3 circumnavigation” sailboat sampling (solid red lines), and the same sampling with added measurement errors. Measurement errors are applied only to the subsampled sailboat fCO_2_ data. Teal lines represent cases with low-end errors (±5 μatm uncertainty or 5 μatm bias), and purple lines represent high-end errors (±10 μatm uncertainty or 10 μatm bias). Random measurement uncertainties are shown as dashed lines (note: they appear on top of each other), while systematic biases are shown as dotted (negative bias) and dash-dotted (positive bias) lines.

Specifically, we consider six scenarios (Fig. 5): (i) a low-end measurement uncertainty of ±5 μatm (best-case scenario); (ii and iii) a constant positive or negative bias of 5 μatm (low-end systematic offset); (iv) a high-end measurement uncertainty of ±10 μatm; and (v and vi) a constant positive or negative bias of 10 μatm (high-end systematic offset). An uncertainty of ±5 μatm is at the lower range of uncertainties observed in the field (*50*, *51*), thus representing a “best-case” estimate, while 10 μatm is at the higher end with a systematic offset representing the “worst-case” scenario of limited calibration and maintenance as well as system limitations.

Sailboat data from “3 circumnavigation” tracks with an associated random measurement uncertainty ranging between ±5 μatm still improve the air-sea CO_2_ flux estimate, presenting a similar picture to unaffected reconstructions, both showing improved reconstructions in the data-sparse period before 2000 and in recent years (see the teal dashed line following the red line in Fig. 5, A to C). This is consistent with other studies examining the effect of random measurement uncertainties, showing that the addition of data can improve air-sea CO_2_ flux estimates, even when those data are affected by high random measurement uncertainties (*17*, *52*).

While adding a negative bias to the measurements worsens the reconstruction as expected (dotted lines in Fig. 5), introducing positive measurement offsets of either 5 or 10 μatm to the sailboat data improves the air-sea CO_2_ flux reconstructions (dash-dotted lines in Fig. 5). The impact of the systematic offsets, both negative and positive, is most pronounced before 1990 in high latitudes (Fig. 5, A and C) and in recent years where biased sailboat data were added starting around 2015 (Fig. 5, A to C), indicating that the bias is not extrapolated in time, but in space. Positive biases lead to reduced CO_2_ uptake in recent years, particularly in the high latitudes (dash-dotted lines in Fig. 5, A and C), and increased outgassing in the tropics (dash-dotted lines in Fig. 5B), whereas negative biases cause the opposite. Similar to (*17*), we also find that Southern Ocean air-sea CO_2_ estimates are most sensitive to measurement errors, particularly measurement biases, with biased estimates diverging from the unbiased estimate around 2005 (Fig. 5C), while estimates in the northern high latitudes and tropics only visibly diverge around 2018/2019 (Fig. 5, A and B).

Notably, positive measurement biases counteract the overestimation of the carbon uptake in high latitudes and underestimation of outgassing in the tropics with “3 circumnavigations” in recent years, bringing the air-sea CO_2_ flux estimates even closer to the model truth (see dash-dotted lines and red solid line compared to black line in Fig. 5, A to C). This shows that the impact of biased observations is more complex than one would expect at first sight. Negative offsets, on the other hand, in “3 circumnavigations” lead to an overestimation of the ocean carbon uptake in high latitudes and underestimation of the outgassing in the tropics especially in recent years.

Focusing on the sailboat tracks alone, we find that both the high and low biased scenarios lead to similar errors, but, as expected, in opposite directions. A 10-μatm positive bias leads to a mean flux bias of 0.14 mol C m^−2^ year^−1^, and a negative measurement bias leads to a flux bias of −0.16 mol C m^−2^ year^−1^. Therefore, the difference likely originates from other places, where the neural network extrapolates the information gained by sailboat tracks. This is visualized in fig. S7. While adding negative biases worsens the reconstruction, adding positive biases actually improves the reconstruction in the Southern Ocean outgassing region, the South Atlantic, and the Equatorial Pacific.

These results indicate that the neural network method used here (*37*) and potentially other machine learning methods are sensitive to systematic biases and their extrapolation. Biased measurements may compensate for missing information (in our case, high-end fCO_2_) elsewhere, improving the reconstruction for the wrong reason (fig. S8). Therefore, even biased observations can improve reconstructions when data availability is limited and data distribution is skewed (fig. S8), as evidenced by the proximity of the dash-dotted and dotted lines to the solid red lines in Fig. 5; however, our analysis shows that this result is strongly linked to the underlying model testbed, the machine learning algorithm, and the direction of the bias.

In conclusion, the improvement in the air-sea CO_2_ flux reconstruction caused by the addition of data from “3 circumnavigations” persists even when the sailboat data contain uncertainties. Although biased data can still contribute to improved overall reconstructions, for the wrong reason through spatial extrapolation, the bias impact becomes substantial when observational constraints are weak: Underobserved regions and data-sparse periods are particularly sensitive. A positive measurement bias may coincidentally shift the reconstruction closer to the model truth. However, negative biases, especially those approaching the upper limit of what is expected from the measurement system, worsen the air-sea CO_2_ flux estimates.

Unlike studies such as (*5*, *53*), which applied bias corrections to the entire underlying observational dataset, our study introduces measurement errors only to a limited subset of sailboat data, similar to (*17*). We show that our method, likely similar to other machine learning approaches, is sensitive to biased predictors and targets; however, the neural network’s sensitivity depends on the extent of biased data, the measurement coverage, and the underlying model used as ground truth.

Although the addition of “3 (unbiased) circumnavigations” improves estimates across all decades by producing a near-constant shift in the reconstructed air-sea CO_2_ flux through time (Fig. 4), the influence of measurement biases is largely confined to the period when these data were sampled (around 2009; Fig. 5). These seemingly contrasting responses arise from different mechanisms by which the neural network integrates information. Adding rare, unbiased, recurring observations enables the model to better represent the underlying statistical distribution of fCO_2_ across the full period, thereby learning a more accurate mean relationship between predictors and target fCO_2_ and improving the reconstruction even in earlier decades, resulting in a near-zero mean bias before 2011 in the fCO_2_ density distribution (fig. S9).

In contrast, introducing systematic measurement biases does not alter the learned relationships several years back in time prior to when they were introduced, but directly affects the estimates where and when the biased data are present. Biased observations influence the reconstruction over time, but the bias signal itself is not propagated back in time; it is mainly expressed during the years when the biased data are introduced, because, in our interpretation, the earlier decades are already well represented in the learned fCO_2_ distribution (fig. S9). Consequently, a systematic bias in the data mainly influences recent years, where these rare observations are added (*54*).

## DISCUSSION

Here, we quantify the improvement in the air-sea CO_2_ flux estimate by adding sailboat observations in an observing system simulation experiment. We find that a commonly used neural network method, using the available real-world sampling scheme, underestimates the model-truth ocean carbon sink, which aligns with findings in (*30*). However, increased sampling with sailboats improves the global estimate and increases the ocean carbon sink from 1982 to the end of 2021, particularly in the North Atlantic and in the Southern Ocean between 40°S and 60°S. For instance, by adding data from “3 circumnavigation” tracks, the global mean air-sea CO_2_ flux density bias decreases from 0.06 to −0.02 mol C m^−2^ year^−1^, and in the Southern Ocean from 0.10 to 0.00 mol C m^−2^ year^−1^.

Despite the observed shift in the mean air-sea CO_2_ flux when adding more data, the overestimated air-sea CO_2_ flux trend persists even with the addition of two additional circumnavigations. We conclude that data from two additional circumnavigations are insufficient to improve the trend, suggesting that the Global Carbon Budget model and data products are likely to continue diverging even with the incorporation of more circumnavigation data (*1*, *46*). Additional data are needed to improve the trend. Currently, the trend in the reconstructions deviates from the model trend. By incorporating more input data over multiple decades that inherently follow the correct trend into the neural network, the output reconstructions are expected to align more closely with the model’s true trend. Thus, we conclude that the inclusion of more sailboat data with trend-consistent information could improve the trend accuracy.

This underscores the need for implementing multidecadal observing strategies. Regular sailboat circumnavigations, such as The Ocean Race and the Vendée Globe, which alternate on a staggered 4-year cycle, present a viable approach. These events ensure a major round-the-world race approximately every 1 to 2 years, thereby providing consistent and comprehensive datasets to refine our understanding and improve the accuracy of reconstructions over extended temporal scales. However, sailboats cannot fully cover seasonal measurement gaps, especially in winter at high latitudes. This further underscores the need to support complementary platforms such as autonomous floats (*27*, *28*) and Saildrones (*32*), which provide year-round data under challenging conditions. Combining these platforms ensures more complete coverage and improves long-term monitoring of air-sea CO_2_ fluxes.

Previous studies have shown that adding data from sailboats and Argo floats to the observing system can significantly affect and improve fCO_2_ and air-sea CO_2_ flux reconstructions, even when the data contain measurement uncertainties, but not if these data are biased (*17*, *52*). Here, we show that adding more data, even if they contain uncertainties, can improve air-sea CO_2_ flux reconstructions and strengthen the overall observing system. This supports previous findings (*55*, *56*) that data quantity can compensate for reduced data quality. However, biased data come with a trade-off. They can still help improve overall reconstructions, but for the wrong reason. Compensating for the lack of high-end fCO_2_ samples in the machine learning training data, biased measurements led to an improved reconstruction in high fCO_2_ regions. As a result, biased data improve global reconstructions while they reduce accuracy regionally where the measurements are actually taken. Such improvements have to be taken with caution as they are likely machine learning– and model-dependent and may not be translatable into the real world, creating a false acceptance for measurement biases. Instead, we show in our study that to interpret reconstructions with biased measurements, it is essential to consider the underlying data density distribution, the model used for the testbed analysis as well as the machine learning background bias (relative to the model truth), and the direction (positive or negative) of any measurement bias before interpreting the machine learning extrapolation, and we highly recommend to consider these in future observing system design studies.

Although the finding that adding new measurements improves the air-sea CO_2_ flux estimates probably holds irrespective of the baseline model choice, the observed magnitude of improvement from increased sailboat sampling is highly model-dependent and could vary with different models. We work under the assumption that HAMOCC represents a known truth for our experiment; however, the HAMOCC model is known to underestimate surface ocean fCO_2_ in the Southern Ocean (*39*) and exhibits an amplified seasonal cycle (*20*). This could affect the results by making the improvements appear more pronounced or less consistent across seasons than they would with a model that accurately captures seasonal variations in fCO_2_, potentially distorting the long-term trend. Furthermore, we only use one neural network method (*37*) on the model data, and different neural network methods might respond differently to data addition. We recommend more extensive analyses in the future including different neural network approaches and different model data, i.e., the products used in the Global Carbon Budget (*1*).

While we studied how underway fCO_2_ measurements from sailboats affect and improve reconstructions, we have yet to identify the specific ocean features responsible for this effect. To determine why these changes and improvements occur, future research should focus on another added value of the data: the high-resolution nature of sailboat data, which resolves important small-scale ocean features driving variability in the air-sea CO_2_ flux [e.g., (*57*)].

The global fCO_2_ measurement coverage is biased toward the Northern Hemisphere, which leads to reconstruction errors; however, the addition of sailboat data provides better data coverage in the Southern Ocean with the potential to improve air-sea CO_2_ flux reconstructions in the future. The frequency of the sailboat races meets the demand for more affordable and innovative platforms (*16*, *58*–*61*). Considering that the Southern Ocean carbon uptake will increase (*62*, *63*) and the observational coverage recently declined [www.socat.info; (*5*)], more observations are needed to closely monitor the ocean carbon sink over multiple decades, and sailboat races provide the opportunity to do that. We conclude that while the addition of sailboat data has the potential to improve air-sea CO_2_ flux reconstructions, particularly in the Southern Ocean, further expansion of the sailboat-based observational network is essential to help minimize discrepancies in Global Carbon Budget sink estimates. Continued efforts to increase the volume and coverage of sailboat measurements will be crucial in refining our understanding of oceanic carbon dynamics.

## MATERIALS AND METHODS

### Datasets and sampling masks

In this study, our primary aim is to quantify the improvement in our air-sea CO_2_ flux estimate caused by adding different sailboat data to our flux reconstructions. To achieve this, we use the model fCO_2_ field as our starting point and apply different subsampling schemes (Fig. 1) to get pseudo-observations. Most of the races took place in the North Atlantic between 2018 and the end of 2021. However, the Antarctic circumnavigation race, Vendée Globe, primarily navigated the Southern Ocean and occurred in 2020/2021, repeating every 4 years. Another notable round-the-world race contributing substantial Southern Ocean data is The Ocean Race, held in 2023 and recurring approximately every 3 years. This study focuses on the period from 1982 to 2021, using The Ocean Race tracks solely to subsample earlier years.

#### Data X—“truth”

Ground truth fCO_2_ values from the HAMOCC model coupled to the ocean general circulation model MPIOM, contributing to Global Carbon Budget simulation at a monthly 1°-by-1° resolution from 1982 until the end of 2021(*1*, *39*–*41*).

The HAMOCC model simulates the oceanic cycles of carbon in the global MPIOM. HAMOCC features biology and inorganic carbon chemistry processes in the water column and sediment (*39*, *64*). Marine primary producers are represented by two state variables: bulk phytoplankton and diazotrophs. The growth of bulk phytoplankton is limited by temperature and light as well as by the availability of nutrients including nitrate, phosphate, and iron linked by constant Redfield ratios across organic compartments. The growth of nitrogen-fixing cyanobacteria is parameterized analogously to that of the bulk phytoplankton, but at a lower rate, and is extended by representing their buoyancy. Zooplankton grazes on bulk phytoplankton, producing particulate organic matter that enters the detritus pool. Opal particles or CaCO_3_ is produced during detritus formation, depending on silicate availability. HAMOCC simulates the upper sediment by 12 biologically active layers and a burial layer to represent the dissolution and decomposition of inorganic and organic matter as well as the diffusion of pore water constituents (*65*). The HAMOCC model has been extensively evaluated in previous studies and successfully used for climate predictions and projections as well as simulating the past climate (*39*–*41*, *66*–*70*).

#### Data A—“existing sailboat”

Subsampled model fCO_2_ (X) generated using the SOCATv2022 sampling scheme, including sailboat data. We used underway fCO_2_ measurement tracks from the International Monohull Open Class Association (IMOCA) 60-foot class sailboats *Seaexplorer-Yacht Club de Monaco* (until 2019 *Malizia*) and *Newrest-Art & Fenêtres* (now *Nexans-Art & Fenêtres*) during offshore sailing and training events from 2018 to 2021. Together, all sailboats collected 161 days of fCO_2_ measurements until the end of 2021.

#### Data B—“without sailboat”

Subsampled model fCO_2_ (X) generated using the SOCATv2022 sampling scheme, excluding sailboat data.

#### Data C—“3 circumnavigations”

Subsampled model fCO_2_ (X) generated using the SOCATv2022 sampling scheme including existing sailboat data and two additional Antarctic circumnavigations (Vendée Globe; 2012/13, 2016/17, and 2020/21) (from which only the latter one is from the existing track and also included in data A; the track of the Vendée Globe 2020/21, where fCO_2_ measurements were taken, was used to subsample the model data in earlier years when Vendée Globe races occurred without fCO_2_ measurements.)

#### Data D—“2 different circumnavigations”

Subsampled model fCO_2_ (X) generated using the SOCATv2022 sampling scheme including sailboat data and two different round-the-world races (Vendée Globe 2020/21 and the preliminary The Ocean Race 2017/18) (for the latter one, we used existing tracks from 2023).

In the following, we added random measurement uncertainties and biases to the subsampled sailboat fCO_2_ data in “3 circumnavigations”:

#### Data E—“3 circumnav + low uncert”

Data C, but with an added random measurement uncertainty ranging from ±5 μatm (lower end of what is expected from the measurement device) to the data from sailboat tracks.

#### Data F—“3 circumnav + 5 offset”

Data C, but with an added +5-μatm measurement offset to the data from sailboat tracks.

#### *Data G—“3 circumnav* − *5 offset”*

Data C, but with an added −5-μatm measurement offset to the data from sailboat tracks.

#### Data H—“3 circumnav + high uncert”

Data C, but with an added random measurement uncertainty ranging from ±10 μatm (higher end of what is expected from the measurement device) to the data from sailboat tracks.

#### Data I—“3 circumnav + 10 offset”

Data C, but with an added +10-μatm measurement offset to the data from sailboat tracks.

#### Data J—“3 circumnav − 10 offset”

Data C, but with an added −10-μatm measurement offset to the data from sailboat tracks.

### The reconstruction of sea surface fCO_2_ and air-sea CO_2_ fluxes

The gaps in all subsampled sea surface fCO_2_ maps (data A to J) were filled using the two-step neural network method called SOM-FFN (*37*). In the first step, an SOM classified the ocean into biogeochemical provinces based on common patterns in predictor variables, including HAMOCC sea-surface temperature, sea-surface salinity, mixed layer depth, and an fCO_2_ climatology (*1*, *39*–*41*). The second step involved an FFN establishing nonlinear relationships between predictors and subsampled fCO_2_ data within each province. The source code for the neural network method can be found in (*71*). Predictors were HAMOCC sea-surface temperature, sea-surface salinity, mixed layer depth, atmospheric CO_2_ concentration, and phytoplankton plus cyanobacteria biomass integrated over a depth of 37 m. This specific depth, limited to the upper ocean biology and excluding the deep chlorophyll maxima, was chosen due to the limitations of equivalently and previously used ocean color observations for case-1 water, which are restricted to the first optical depth below 40 m (*72*). The MPIOM-HAMOCC model output can be found in (*73*). Based on the reconstructed fCO_2_ data A to J and model truth X, we computed flux estimates A to J and X by applying a bulk gas transfer formulation with a quadratic relationship between wind speed and transfer velocity to the reconstructed maps (A to J), as well as the model truth X (*37*, *49*). The mean gas transfer was standardized to a global average rate of 16.5 cm hour^−1^ (*74*). While the neural network reconstructed fCO_2_ closely matches the model fCO_2_, the reconstructed air-sea CO_2_ flux differs from the model flux. This discrepancy arises because of the bulk gas transfer parametrization with a standardized mean gas transfer velocity of 16.5 cm hour^−1^, which is not ideal for the model fCO_2_. Acknowledging that our primary focus is not on realistically estimating the air-sea CO_2_ flux but rather on assessing the impact of different sampling schemes on the flux estimate, we decided to eliminate this uncertainty by applying the same parametrization on the model fCO_2_ (*49*). This allowed us to establish a comparable “original” flux model truth for our analysis.

We focus both on the air-sea CO_2_ flux and the fCO_2_ because of the high sensitivity of air-sea CO_2_ flux estimates to errors in fCO_2_. In high wind regions, small fCO_2_ errors can lead to large flux errors due to high gas transfer velocity, while the same fCO_2_ error in low wind regions results in smaller flux errors. Furthermore, the direction of the fCO_2_ error improvement (negative or positive) significantly affects the air-sea CO_2_ flux estimate. A small negative improvement in fCO_2_ can either decrease or increase the flux estimate depending on whether the ocean fCO_2_ is greater or less than the atmospheric fCO_2_, respectively. Similarly, a small positive improvement can increase or decrease the flux estimate based on the same conditions. By focusing on both estimates, we ensure a comprehensive assessment, minimizing the risk of overlooking critical factors that could skew the air-sea CO_2_ flux estimates.

In this study, we define the air-sea CO_2_ flux density as the instantaneous flux into or out of the ocean [in units of moles of carbon per square meter per year, (mol C m^−2^ year^−1^)], where positive flux indicates outgassing and negative flux indicates uptake (fig. S5), generally aligning its direction with fCO_2_. In contrast, the air-sea CO_2_ flux—as well as the ocean carbon sink when referring to a negative integrated signal—is quantified as the spatially integrated flux over a given area [in petagrams of carbon, (PgC)] and typically follows the opposite direction.

### Statistical analyses

We exclude regions with a climatological maximum sea-ice concentration greater than 50% from most of our analysis as sparse observations (*4*), and the influence of seasonal changes in sea-ice coverage introduces high uncertainties into the neural network reconstruction in that region (*11*, *12*, *45*, *75*) and due to the model’s less realistic representation of the high latitudes (*39*). The ice zone in the Southern Ocean coincides with the high uptake region south of ~60°S (fig. S5).

#### Neural network performance evaluation

To evaluate the performance of the neural network in reconstructing the HAMOCC fCO_2_, we use probability density functions and calculate the BD (*43*), which measures the similarity between the probability density functions of the reconstruction and the original model fCO_2_. Lower BD values indicate a higher degree of similarity between the probability density functions. We further compare the detectable signal in the air-sea CO_2_ flux estimate caused by the addition of sailboat data in our subsampled model data to the signal in observation data from a previous study using a signal-to-noise detection described in (*17*).

#### Improvement quantification

We used a Monte Carlo approach, generating 10- to 40-member ensembles for each of the 10 subsampling scenarios. For scenarios A (“existing sailboat”), B (“without sailboat”), C (“3 circumnavigations”), D (“2 different circumnavigations”), E (“3 circumnavigations + low measurement uncertainty”), and F (“3 circumnavigations + positive 5 μatm offset”), we generated 40 ensemble members each. For scenarios G (“3 circumnavigations − 5 μatm offset”), H (“3 circumnavigations + high measurement uncertainty”), I (“3 circumnavigations + 10 μatm offset”), and J (“3 circumnavigations − 10 μatm offset”), we generated 10 ensemble members each, as the standard deviation across 10 versus 40 ensembles differs only slightly and using fewer members allows for more computationally efficient analysis.

We generated the ensembles by varying training and validation dataset splits to enhance the reliability of our air-sea CO_2_ flux estimates and identify potential random errors caused by the sensitivity of the neural-network approach to different subsets of the data. The ensemble mean served as the best estimate for each sampling scenario.

The bias is calculated as the mean of the reconstruction R (i.e., A, B, and C) minus the model truth (X): bias = mean(R) − mean(X) and measures the over- and underestimation in the reconstructions over different time periods. Values near zero not only indicate a good reconstruction but could also indicate that positive and negative differences cancel out. We calculated the mean bias as well as the median bias to minimize the impact of regional outliers caused by regions of high uncertainty (Fig. 2).

## Supplementary Materials

### This PDF file includes:

Figs. S1 to S9 References
