# Supplementary material for: Improved air-sea CO2 flux estimates from sailboat measurements
Source: Sci Adv. 2026 Jan 9;12(2):eadz1502. doi: 10.1126/sciadv.adz1502 (PMC12787523; doi:10.1126/sciadv.adz1502)
Supplement: Supplementary file 1 — Figs. S1 to S9 References [file sciadv.adz1502_sm.pdf]

Science Advances
AAAS

Supplementary Materials for

Improved air-sea CO_2 flux estimates from sailboat measurements

Jacqueline Behncke *et al.*

Corresponding author: Jacqueline Behncke, jacqueline.behncke@mpimet.mpg.de

Sci. Adv. **12**, eadz1502 (2026)
DOI: 10.1126/sciadv.adz1502

This PDF file includes:

Figs. S1 to S9
References

Fig. S1. Reconstruction Uncertainty Assessment

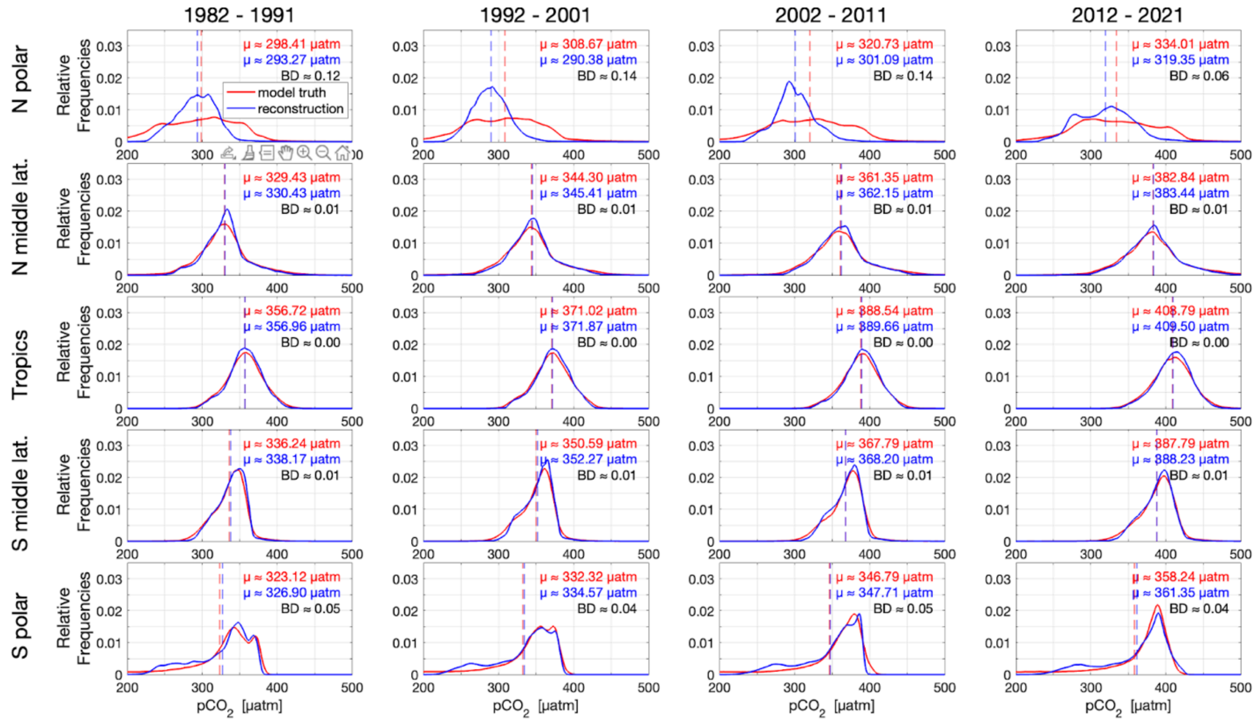

Kernel Density estimate of fCO_2 data distributions of model truth fCO_2 (X; red) and subsampled and NN-reconstructed fCO_2 (A "existing sailboat"; blue) for different regions and time periods. Regions with a climatological maximum sea-ice concentration greater than 50% are included. Latitudinal zones are divided into increments of 30 degrees, ranging from 90°N to 90°S. The time periods were chosen to demonstrate how the addition of sailboat data impacts the fCO_2 and, by extension, the air-sea CO_2 flux across the entire time period, independently of the specific years in which data were added.

Fig. S2 Latitudinal Bias of fCO_2 Reconstructions

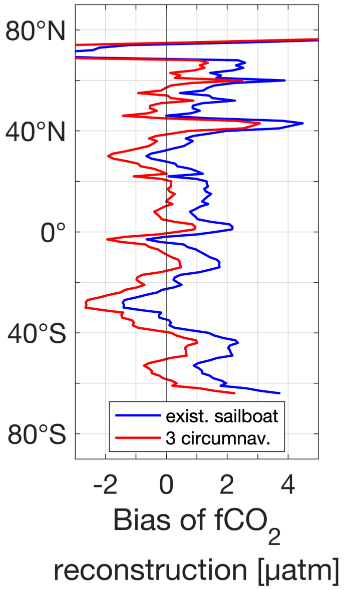

Latitudinal Bias of the fCO_2 Reconstructions "*existing sailboat*" and "*3 circumnavigations*" compared to model truth.

Fig. S3. Effect of "*3 Circumnavigations*" on Southern Ocean fCO_2 Distribution (2002-2021)

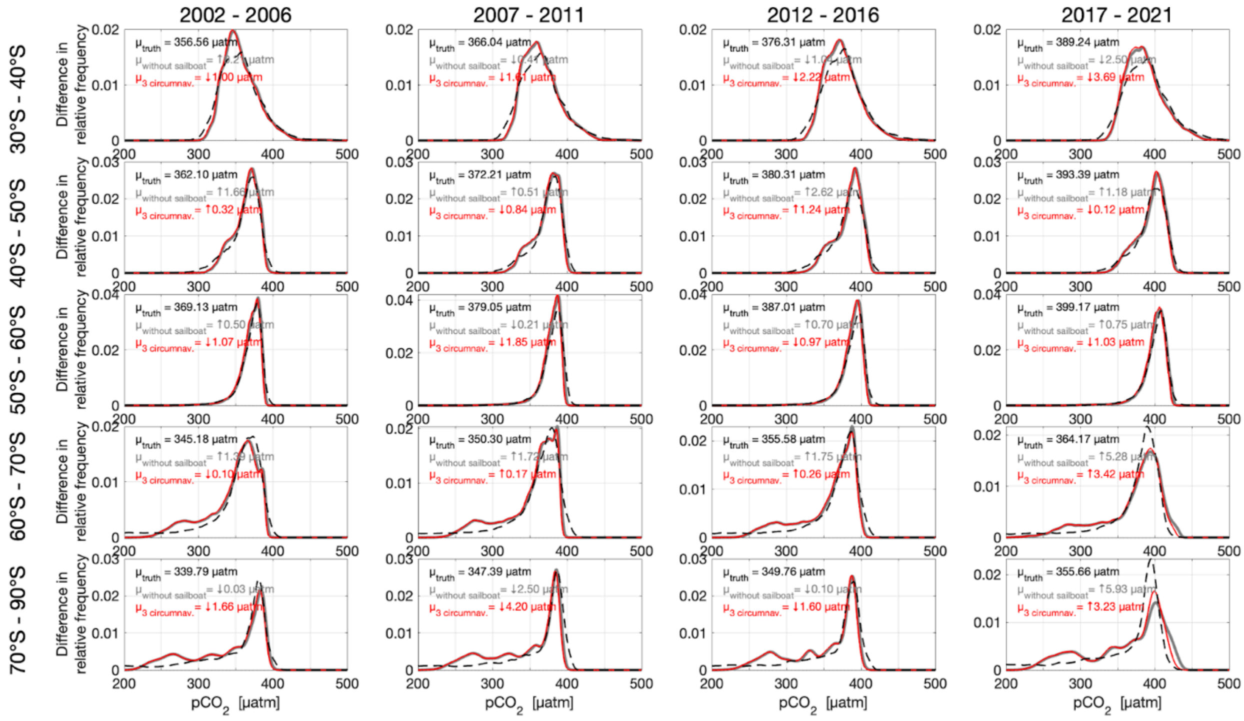

Difference between the data distribution of fCO_2 estimates based on sampling scheme B "without sailboat" (gray) and C "3 circumnavigations" (red) in the Southern Ocean between 2002-2021. *Regions with a climatological maximum sea-ice concentration greater than 50% are included. Additional sailboat data reduce the fCO_2 estimate in the Southern Ocean (see change from gray line to red line).*

Fig. S4. Comparing the Impact of Observational and Model-Based Sailboat Data Addition on Air-Sea CO_2 Flux Estimates

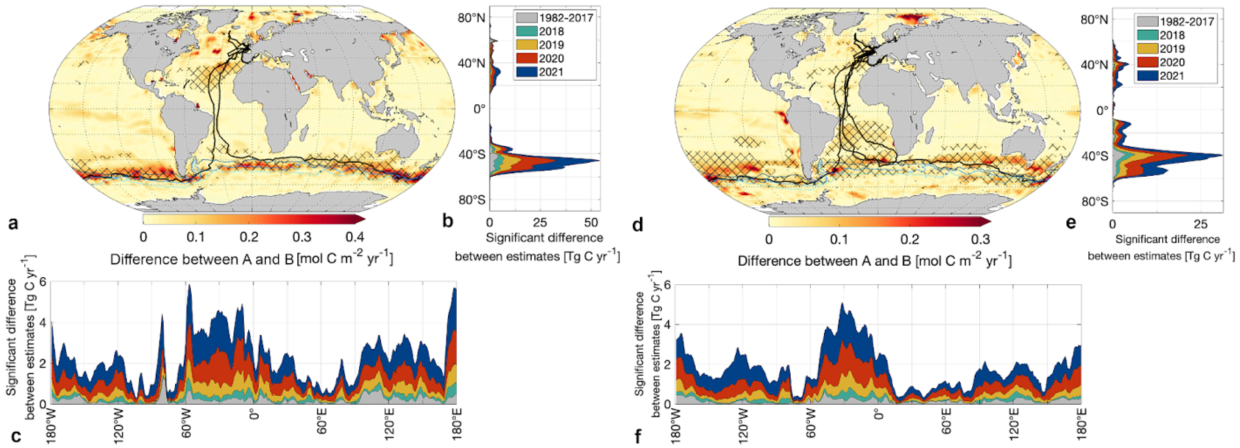

The detectable change in the air-sea CO_2 flux estimate caused by adding the existing sailboat data to the reconstructions. a-c) observation-based reconstructions (17) and d-f) (subsampled) model-based reconstructions. a) and d) Maps show the detectable change in the air-sea CO_2 flux estimate caused by adding the existing sailboat data to the reconstructions averaged over November 2020 to January 2021 (the time of the Vendée Globe circumnavigation). Hatching indicates significant differences. b,c) and e,f) Significant differences between air-sea CO2 flux estimates per year and (b, e) latitude and (c, f) longitude. Blue lines from north to south: Northern Boundary, Subantarctic Front, Polar Front (76). Note the different ranges on the axes. a-c) includes tracks from a single sailboat (17), while d-f) include tracks from all sailboats measuring fCO_2 and contributing to SOCAT (4) up until the end of 2021 (used in this study).

Fig. S5. Reconstructed Air-Sea CO_2 Flux Density Based on SOCAT-Sampling

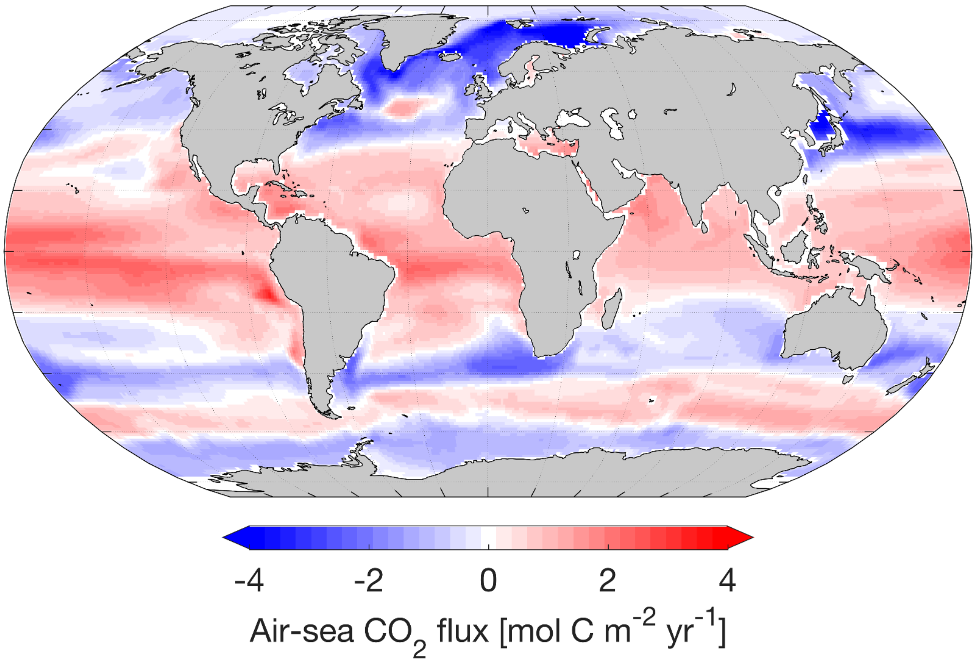

Reconstructed air-sea CO_2 flux density based on SOCAT-sampling.

Fig. S6. Annual Time Series of Mean Absolute Errors

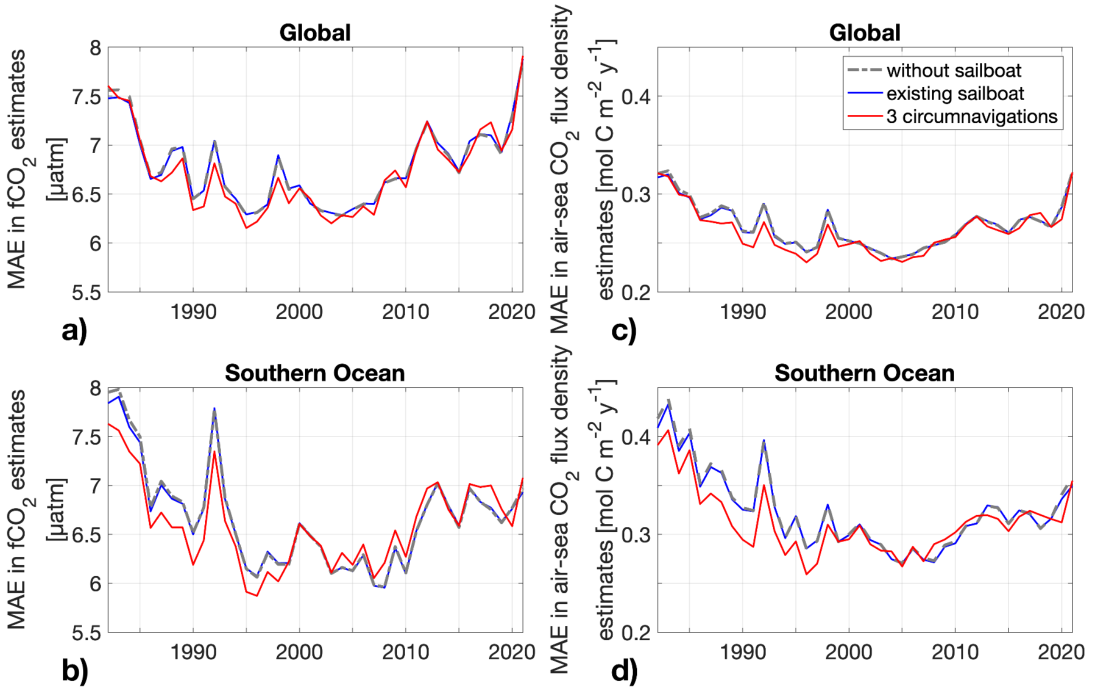

Annual time series of Mean Absolute Errors (MAE) in reconstructed a-b) fCO_2 (left column) and c-d) air-sea CO_2 flux density (right column).

Fig. S7: Spatial Difference in the MAE of Reconstructed Air-Sea CO_2 Fluxes between Biased (±10 µatm) and Unbiased "*3 Circumnavigation*" Scenarios.

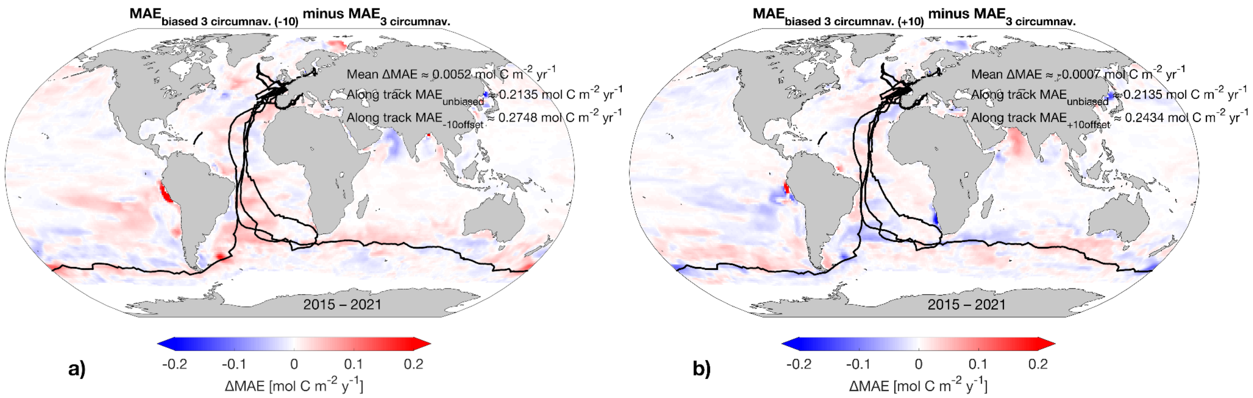

Spatial difference in the MAE of reconstructed air-sea CO_2 fluxes between biased (±10 µatm) and unbiased "3 circumnavigation" scenarios. ΔMAE was calculated as $MAE_{\textbf{biased\_reconstruction}}$ − $MAE_{\textbf{unbiased\_reconstruction}}$. *Positive (red) values indicate regions where the unbiased run yielded lower reconstruction errors (i.e., performed better), while negative (blue) values indicate areas where the biased run performed better. Track-averaged MAE and bias values are shown to the right of each map and indicate that the addition of biased observations systematically increases both the bias and MAE along the sailboat track.a) −10 µatm bias scenario; b) +10 µatm bias scenario.*

Fig. S8: Data Density Distribution of Model Truth fCO_2, Unbiased "*3 Circumnavigations*", and +10 µatm "*Biased 3 Circumnavigations*" South of 30°S Between 2015 and 2021.

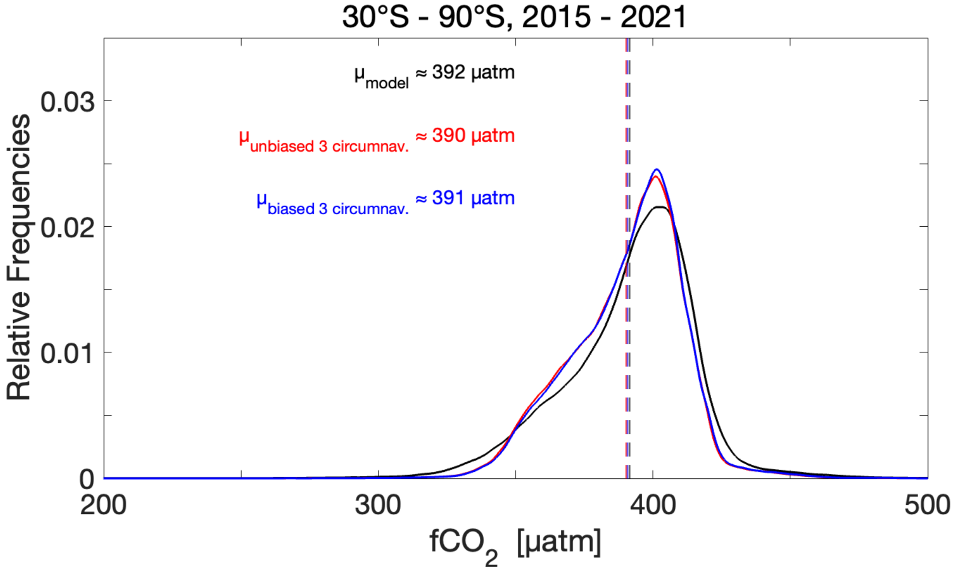

Kernel density estimate of data distribution of model truth fCO_2, unbiased *"3 circumnavigations*", and +10 µatm "*biased 3 circumnavigations*" south of 30°S between 2015 and 2021. Regions with a climatological maximum sea-ice concentration greater than 50% are excluded. "*Biased 3 circumnavigations*" (blue) shift the data distribution closer to the model truth (black) than unbiased "*3 circumnavigations*" (red).

Figure S9: Data Density Distributions of fCO_2 for the Model Truth Case, the "*Existing Sailboat"* Case, the "*3 Circumnavigations"* Case and the "*Biased (+10 µatm) Circumnavigations"* Case for Four Different Decades from 1982 Through 2021.

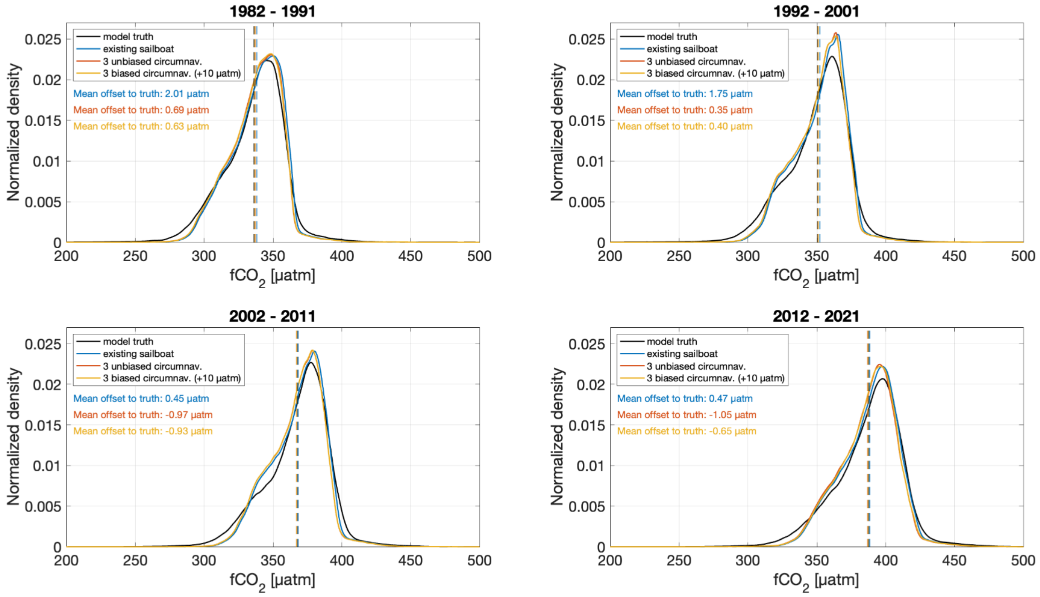

Density distributions (PDF) of the mean sea surface fCO_2 south of 30°S for the perfect model case, the "*existing sailboat"* case, the "*3 circumnavigations"* case and the "*biased (+10 µatm) circumnavigations"* case for four different decades from 1982 through 2021. *Figure labels indicate the experiment, whereas colored text contains the difference between the PDF mean and the model truth in the experiments. Regions with a climatological maximum sea-ice concentration greater than 50% are excluded.*

REFERENCES

1. P. Friedlingstein, M. O'Sullivan, M. W. Jones, R. M. Andrew, J. Hauck, P. Landschützer, C. Le Quéré, H. Li, I. T. Luijkx, A. Olsen, G. P. Peters, W. Peters, J. Pongratz, C. Schwingshackl, S. Sitch, J. G. Canadell, P. Ciais, R. B. Jackson, S. R. Alin, A. Arneth, V. Arora, N. R. Bates, M. Becker, N. Bellouin, C. F. Berghoff, H. C. Bittig, L. Bopp, P. Cadule, K. Campbell, M. A. Chamberlain, N. Chandra, F. Chevallier, L. P. Chini, T. Colligan, J. Decayeux, L. M. Djeutchouang, X. Dou, C. Duran Rojas, K. Enyo, W. Evans, A. R. Fay, R. A. Feely, D. J. Ford, A. Foster, T. Gasser, M. Gehlen, T. Gkritzalis, G. Grassi, L. Gregor, N. Gruber, Ö. Gürses, I. Harris, M. Hefner, J. Heinke, G. C. Hurtt, Y. Iida, T. Ilyina, A. R. Jacobson, A. K. Jain, T. Jarníková, A. Jersild, F. Jiang, Z. Jin, E. Kato, R. F. Keeling, K. Klein Goldewijk, J. Knauer, J. I. Korsbakken, X. Lan, S. K. Lauvset, N. Lefèvre, Z. Liu, J. Liu, L. Ma, S. Maksyutov, G. Marland, N. Mayot, P. C. McGuire, N. Metzl, N. M. Monacci, E. J. Morgan, S.-I. Nakaoka, C. Neill, Y. Niwa, T. Nützel, L. Olivier, T. Ono, P. I. Palmer, D. Pierrot, Z. Qin, L. Resplandy, A. Roobaert, T. M. Rosan, C. Rödenbeck, J. Schwinger, T. L. Smallman, S. M. Smith, R. Sospedra-Alfonso, T. Steinhoff, Q. Sun, A. J. Sutton, R. Séférian, S. Takao, H. Tatebe, H. Tian, B. Tilbrook, O. Torres, E. Tourigny, H. Tsujino, F. Tubiello, G. van der Werf, R. Wanninkhof, X. Wang, D. Yang, X. Yang, Z. Yu, W. Yuan, X. Yue, S. Zaehle, N. Zeng, J. Zeng, Global carbon budget 2024. *Earth Syst. Sci. Data* **17**, 965–1039 (2025).

2. N. Gruber, D. C. E. Bakker, T. DeVries, L. Gregor, J. Hauck, P. Landschützer, G. A. McKinley, J. D. Müller, Trends and variability in the ocean carbon sink. *Nat. Rev. Earth Environ.* **4**, 119–134 (2023).

3. C. Le Quéré, C. Rödenbeck, E. T. Buitenhuis, T. J. Conway, R. Langenfelds, A. Gomez, C. Labuschagne, M. Ramonet, T. Nakazawa, N. Metzl, N. Gillett, M. Heimann, Saturation of the Southern Ocean CO_2 sink due to recent climate change. *Science* **316** (5832), 1735–1738 (2007).

4. D. C. E. Bakker, B. Pfeil, C. S. Landa, N. Metzl, K. M. O’Brien, A. Olsen, K. Smith, C. Cosca, S. Harasawa, S. D. Jones, S. Nakaoka, Y. Nojiri, U. Schuster, T. Steinhoff, C. Sweeney, T. Takahashi, B. Tilbrook, C. Wada, R. Wanninkhof, S. R. Alin, C. F. Balestrini, L. Barbero, N. R. Bates, A. A. Bianchi, F. Bonou, J. Boutin, Y. Bozec, E. F. Burger, W.-J. Cai, R. D. Castle, L. Chen, M. Chierici, K. Currie, W. Evans, C. Featherstone, R. A. Feely, A. Fransson, C. Goyet, N.

Greenwood, L. Gregor, S. Hankin, N. J. Hardman-Mountford, J. Harlay, J. Hauck, M. Hoppema, M. P. Humphreys, C. W. Hunt, B. Huss, J. S. P. Ibánhez, T. Johannessen, R. Keeling, V. Kitidis, A. Körtzinger, A. Kozyr, E. Krasakopoulou, A. Kuwata, P. Landschützer, S. K. Lauvset, N. Lefèvre, C. Lo Monaco, A. Manke, J. T. Mathis, L. Merlivat, F. J. Millero, P. M. S. Monteiro, D. R. Munro, A. Murata, T. Newberger, A. M. Omar, T. Ono, K. Paterson, D. Pearce, D. Pierrot, L. L. Robbins, S. Saito, J. Salisbury, R. Schlitzer, B. Schneider, R. Schweitzer, R. Sieger, I. Skjelvan, K. F. Sullivan, S. C. Sutherland, A. J. Sutton, K. Tadokoro, M. Telszewski, M. Tuma, S. M. A. C. van Heuven, D. Vandemark, B. Ward, A. J. Watson, S. Xu, A multi-decade record of high-quality fCO_2 data in version 3 of the Surface Ocean CO_2 Atlas (SOCAT). *Earth Syst. Sci. Data* **8** (2), 383–413 (2016).

5. Y. Dong, D. C. E. Bakker, P. Landschützer, Accuracy of ocean CO_2 uptake estimates at a risk by a reduction in the data collection. *Geophys. Res. Lett.* **51**, e2024GL108502 (2024).

6. Z.-P. Jiang, J. Yuan, S. E. Hartman, W. Fan, Enhancing the observing capacity for the surface ocean by the use of volunteer observing ship. *Acta Oceanol. Sin.* **38**, 114–120 (2019).

7. B. Pfeil, A. Olsen, D. C. E. Bakker, S. Hankin, H. Koyuk, A. Kozyr, J. Malczyk, A. Manke, N. Metzl, C. L. Sabine, J. Akl, S. R. Alin, N. Bates, R. G. J. Bellerby, A. Borges, J. Boutin, P. J. Brown, W.-J. Cai, F. P. Chavez, A. Chen, C. Cosca, A. J. Fassbender, R. A. Feely, M. González-Dávila, C. Goyet, B. Hales, N. Hardman-Mountford, C. Heinze, M. Hood, M. Hoppema, C. W. Hunt, D. Hydes, M. Ishii, T. Johannessen, S. D. Jones, R. M. Key, A. Körtzinger, P. Landschützer, S. K. Lauvset, N. Lefèvre, A. Lenton, A. Lourantou, L. Merlivat, T. Midorikawa, L. Mintrop, C. Miyazaki, A. Murata, A. Nakadate, Y. Nakano, S. Nakaoka, Y. Nojiri, A. M. Omar, X. A. Padin, G.-H. Park, K. Paterson, F. F. Perez, D. Pierrot, A. Poisson, A. F. Ríos, J. M. Santana-Casiano, J. Salisbury, V. V. S. S. Sarma, R. Schlitzer, B. Schneider, U. Schuster, R. Sieger, I. Skjelvan, T. Steinhoff, T. Suzuki, T. Takahashi, K. Tedesco, M. Telszewski, H. Thomas, B. Tilbrook, J. Tjiputra, D. Vandemark, T. Veness, R. Wanninkhof, A. J. Watson, R. Weiss, C. S. Wong, H. Yoshikawa-Inoue, A Uniform, Quality controlled Surface Ocean CO_2 Atlas (SOCAT). *Earth Syst. Sci. Data* **5**, 125–143 (2013).

8. C. L. Sabine, S. Hankin, H. Koyuk, D. C. E. Bakker, B. Pfeil, A. Olsen, N. Metzl, A. Kozyr, A. Fassbender, A. Manke, J. Malczyk, J. Akl, S. R. Alin, R. G. J. Bellerby, A. Borges, J. Boutin, P. J. Brown, W.-J. Cai, F. P. Chavez, A. Chen, C. Cosca, R. A. Feely, M. González-Dávila, C. Goyet, N. Hardman-Mountford, C. Heinze, M. Hoppema, C. W. Hunt, D. Hydes, M. Ishii, T. Johannessen, R. M. Key, A. Körtzinger, P. Landschützer, S. K. Lauvset, N. Lefèvre, A. Lenton, A. Lourantou, L. Merlivat, T. Midorikawa, L. Mintrop, C. Miyazaki, A. Murata, A. Nakadate, Y. Nakano, S. Nakaoka, Y. Nojiri, A. M. Omar, X. A. Padin, G.-H. Park, K. Paterson, F. F. Perez, D. Pierrot, A. Poisson, A. F. Ríos, J. Salisbury, J. M. Santana-Casiano, V. V. S. S. Sarma, R. Schlitzer, B. Schneider, U. Schuster, R. Sieger, I. Skjelvan, T. Steinhoff, T. Suzuki, T. Takahashi, K. Tedesco, M. Telszewski, H. Thomas, B. Tilbrook, D. Vandemark, T. Veness, A. J. Watson, R. Weiss, C. S. Wong, H. Yoshikawa-Inoue, Surface Ocean CO_2 Atlas (SOCAT) gridded data products. *Earth Syst. Sci. Data* **5**, 145–153 (2013).

9. L. Gloege, G. A. McKinley, P. Landschützer, A. R. Fay, T. L. Frölicher, J. C. Fyfe, T. Ilyina, S. Jones, N. S. Lovenduski, K. B. Rodgers, S. Schlunegger, Y. Takano, Quantifying errors in observationally based estimates of ocean carbon sink variability. *Global Biogeochem. Cycles* **35**, e2020GB006788 (2021).

10. J. Hauck, C. Nissen, P. Landschützer, C. Rödenbeck, S. Bushinsky, A. Olsen, Sparse observations induce large biases in estimates of the global ocean CO_2 sink: An ocean model subsampling experiment. *Philos. Trans. R. Soc. A* **381**, 20220063 (2023).

11. A. Jersild, P. Landschützer, A spatially explicit uncertainty analysis of the air-sea CO_2 flux from observations. *Geophys. Res. Lett.* **51**, e2023GL106636 (2024).

12. D. J. Ford, J. Blannin, J. Watts, A. J. Watson, P. Landschützer, A. Jersild, J. D. Shutler, A comprehensive analysis of air-sea CO_2 flux uncertainties constructed from surface ocean data products. *Global Biogeochem. Cycles* **38**, e2024GB008188 (2024).

13. T. L. Frölicher, J. L. Sarmiento, D. J. Paynter, J. P. Dunne, J. P. Krasting, M. Winton, Dominance of the Southern Ocean in anthropogenic carbon and heat uptake in CMIP5 models. *J. Climate* **28**, 862–886 (2015).

14. P. Landschützer, N. Gruber, D. C. E. Bakker, Decadal variations and trends of the global ocean carbon sink. *Global Biogeochem. Cycles* **30**, 1396–1417 (2016).

15. R. G. Williams, A. J. S. Meijers, V. M. Roussenov, A. Katavouta, P. Ceppi, J. P. Rosser, P. Salvi, Asymmetries in the Southern Ocean contribution to global heat and carbon uptake. *Nat. Clim. Chang.* **14**, 823–831 (2024).

16. L. Gregor, A. D. Lebehot, S. Kok, P. M. S. Monteiro, A comparative assessment of the uncertainties of global surface-ocean CO_2 estimates using a machine learning ensemble (CSIR-ML6 Version 2019a)—Have we hit the wall? *Geosci. Model Dev. Discuss.* **12**, 5113–5136 (2019).

17. J. Behncke, P. Landschützer, T. Tanhua, A detectable change in the air-sea CO_2 flux estimate from sailboat measurements. *Sci. Rep.* **14**, 3345 (2024).

18. P. Landschützer, T. Tanhua, J. Behncke, L. Keppler, Sailing through the southern seas of air–sea CO_2 flux uncertainty. *Philos. Trans. R. Soc. A* **381**, 20220064 (2023).

19. F. F. Pérez, M. Becker, N. Goris, M. Gehlen, M. López-Mozos, J. Tjiputra, A. Olsen, J. D. Müller, I. E. Huertas, T. T. T. Chau, V. Cainzos, A. Velo, G. Benard, J. Hauck, N. Gruber, R. Wanninkhof, An assessment of CO_2 storage and sea-air fluxes for the Atlantic Ocean and mediterranean sea between 1985 and 2018. *Global Biogeochem. Cycles* **38**, e2023GB007862 (2024).

20. P. Rustogi, P. Landschützer, S. Brune, J. Baehr, The impact of seasonality on the annual air-sea carbon flux and its interannual variability. *NPJ Clim. Atmos. Sci.* **6**, 66 (2023).

21. C. Ostle, P. Landschützer, M. Edwards, M. Johnson, S. Schmidtko, U. Schuster, A. J. Watson, C. Robinson, Multidecadal changes in biology influence the variability of the North Atlantic carbon sink. *Environ. Res. Lett.* **17**, 114056 (2022).

22. X. Yang, C. A. Wynn-Edwards, P. G. Strutton, E. H. Shadwick, Drivers of air-sea CO_2 flux in the subantarctic zone revealed by time series observations. *Global Biogeochem. Cycles* **38**, e2023GB007766 (2024).

23. F. P. Chavez, J. Sevadjian, C. Wahl, J. Friederich, G. E. Friederich, Measurements of pCO_2 and pH from an autonomous surface vehicle in a coastal upwelling system. *Deep Sea Res. Part II Top. Stud. Oceanogr.* **151**, 137–146 (2018).

24. T. Daniel, J. Manley, N. Trenaman, The wave glider: Enabling a new approach to persistent ocean observation and research. *Ocean Dyn.* **61**, 1509–1520 (2011).

25. P. M. S. Monteiro, L. Gregor, M. Lévy, S. Maenner, C. L. Sabine, S. Swart, Intraseasonal variability linked to sampling alias in air-sea CO_2 fluxes in the Southern Ocean. *Geophys. Res. Lett.* **42**, 8507–8514 (2015).

26. S.-A. Nicholson, D. B. Whitt, I. Fer, M. D. du Plessis, A. D. Lebéhot, S. Swart, A. J. Sutton, P. M. S. Monteiro, Storms drive outgassing of CO_2 in the subpolar Southern Ocean. *Nat. Commun.* **13**, 158 (2022).

27. S. M. Bushinsky, P. Landschützer, C. Rödenbeck, A. R. Gray, D. Baker, M. R. Mazloff, L. Resplandy, K. S. Johnson, J. L. Sarmiento, Reassessing Southern Ocean air-sea CO_2 flux estimates with the addition of biogeochemical float observations. *Global Biogeochem. Cycles* **33**, 1370–1388 (2019).

28. A. R. Gray, K. S. Johnson, S. M. Bushinsky, S. C. Riser, J. L. Russell, L. D. Talley, R. Wanninkhof, N. L. Williams, J. L. Sarmiento, Autonomous biogeochemical floats detect significant carbon dioxide outgassing in the high-latitude Southern Ocean. *Geophys. Res. Lett.* **45**, 9049–9057 (2018).

29. N. L. Williams, L. W. Juranek, R. A. Feely, K. S. Johnson, J. L. Sarmiento, L. D. Talley, A. G. Dickson, A. R. Gray, R. Wanninkhof, J. L. Russell, S. C. Riser, Y. Takeshita, Calculating surface ocean pCO_2 from biogeochemical Argo floats equipped with pH: An uncertainty analysis. *Global Biogeochem. Cycles* **31**, 591–604 (2017).

30. T. H. Heimdal, G. A. McKinley, A. J. Sutton, A. R. Fay, L. Gloege, Assessing improvements in global ocean pCO_2 machine learning reconstructions with Southern Ocean autonomous sampling. *Biogeosciences* **21**, 2159–2176 (2024).

31. S. Nickford, J. B. Palter, L. Mu, The importance of contemporaneous wind and $p$$CO_2$ measurements for regional air-sea CO_2 flux estimates. *J. Geophys. Res. Oceans* **129**, e2023JC020744 (2024).

32. A. J. Sutton, N. L. Williams, B. Tilbrook, Constraining Southern Ocean CO_2 flux uncertainty using uncrewed surface vehicle observations. *Geophys. Res. Lett.* **48**, e2020GL091748 (2021).

33. A. Denvil-Sommer, M. Gehlen, M. Vrac, Observation system simulation experiments in the Atlantic Ocean for enhanced surface ocean pCO_2 reconstructions. *Ocean Sci.* **17**, 1011–1030 (2021).

34. L. M. Djeutchouang, N. Chang, L. Gregor, M. Vichi, P. M. S. Monteiro, The sensitivity of pCO_2 reconstructions to sampling scales across a Southern Ocean sub-domain: A semi-idealized ocean sampling simulation approach. *Biogeosciences* **19**, 4171–4195 (2022).

35. S. Henson, K. Bisson, M. L. Hammond, A. Martin, C. Mouw, A. Yool, Effect of sampling bias on global estimates of ocean carbon export. *Environ. Res. Lett.* **19**, 024009 (2024).

36. J. Yun, J. Liu, K. W. Bowman, L. Resplandy, D. Carroll, The potential of the orbiting carbon observatory-2 column CO_2 measurements to constrain air-sea CO_2 fluxes. *ESS Open Arch. eprints* **389**, 172252933.38918992 (2024).

37. P. Landschützer, N. Gruber, D. C. E. Bakker, U. Schuster, S. Nakaoka, M. R. Payne, T. P. Sasse, J. Zeng, A neural network-based estimate of the seasonal to inter-annual variability of the Atlantic Ocean carbon sink. *Biogeosciences* **10**, 7793–7815 (2013).

38. Y. Wei, S. T. Gille, M. R. Mazloff, V. Tamsitt, S. Swart, D. Chen, L. Newman, Optimizing mooring placement to constrain Southern Ocean air–sea fluxes. *J. Atmos. Oceanic Tech.* **37**, 1365–1385 (2020).

39. T. Ilyina, K. D. Six, J. Segschneider, E. Maier-Reimer, H. Li, I. Núñez-Riboni, Global ocean biogeochemistry model HAMOCC: Model architecture and performance as component of the MPI-Earth system model in different CMIP5 experimental realizations. *J. Adv. Model. Earth Syst.* **5**, 287–315 (2013).

40. T. Mauritsen, J. Bader, T. Becker, J. Behrens, M. Bittner, R. Brokopf, V. Brovkin, M. Claussen, T. Crueger, M. Esch, I. Fast, S. Fiedler, D. Fläschner, V. Gayler, M. Giorgetta, D. S. Goll, H. Haak, S. Hagemann, C. Hedemann, C. Hohenegger, T. Ilyina, T. Jahns, D. Jimenéz-de-la-Cuesta, J. Jungclaus, T. Kleinen, S. Kloster, D. Kracher, S. Kinne, D. Kleberg, G. Lasslop, L. Kornblueh, J. Marotzke, D. Matei, K. Meraner, U. Mikolajewicz, K. Modali, B. Möbis, W. A. Müller, J. E. M. S. Nabel, C. C. W. Nam, D. Notz, S.-S. Nyawira, H. Paulsen, K. Peters, R. Pincus, H. Pohlmann, J. Pongratz, M. Popp, T. J. Raddatz, S. Rast, R. Redler, C. H. Reick, T. Rohrschneider, V. Schemann, H. Schmidt, R. Schnur, U. Schulzweida, K. D. Six, L. Stein, I. Stemmler, B. Stevens, J.-S. von Storch, F. Tian, A. Voigt, P. Vrese, K.-H. Wieners, S. Wilkenskjeld, A. Winkler, E. Roeckner, Developments in the MPI-M Earth system model version 1.2 (MPI-ESM1.2) and its response to increasing CO_2. *J. Adv. Model. Earth Syst.* **11**, 998–1038 (2019).

41. H. Paulsen, T. Ilyina, K. D. Six, I. Stemmler, Incorporating a prognostic representation of marine nitrogen fixers into the global ocean biogeochemical model HAMOCC. *J. Adv. Model. Earth Syst.* **9**, 438–464 (2017).

42. P. Landschützer, G. G. Laruelle, A. Roobaert, P. Regnier, A uniform pCO_2 climatology combining open and coastal oceans. *Earth Syst. Sci. Data* **12**, 2537–2553 (2020).

43. A. Bhattacharyya, On a measure of divergence between two statistical populations defined by their probability distributions. *Bull. Calcutta Math. Soc.* **35**, 99–109 (1943).

44. A. Roobaert, G. G. Laruelle, P. Landschützer, N. Gruber, L. Chou, P. Regnier, The spatiotemporal dynamics of the sources and sinks of CO_2 in the global coastal ocean. *Global Biogeochem. Cycles* **33**, 1693–1714 (2019).

45. A. Roobaert, P. Regnier, P. Landschützer, G. G. Laruelle, A novel sea surface pCO_2-product for the global coastal ocean resolving trends over 1982−2020. *Earth Syst. Sci. Data* **16**, 421–441 (2024).

46. T. DeVries, K. Yamamoto, R. Wanninkhof, N. Gruber, J. Hauck, J. D. Müller, L. Bopp, D. Carroll, B. Carter, T.-T.-T. Chau, S. C. Doney, M. Gehlen, L. Gloege, L. Gregor, S. Henson, J.

H. Kim, Y. Iida, T. Ilyina, P. Landschützer, C. Le Quéré, D. Munro, C. Nissen, L. Patara, F. F. Pérez, L. Resplandy, K. B. Rodgers, J. Schwinger, R. Séférian, V. Sicardi, J. Terhaar, J. Triñanes, H. Tsujino, A. Watson, S. Yasunaka, J. Zeng, Magnitude, trends, and variability of the global ocean carbon sink from 1985 to 2018. *Glob. Biogeochem. Cycles* **37**, e2023GB007780 (2023).

47. J. Hauck, M. Zeising, C. Le Quéré, N. Gruber, D. C. E. Bakker, L. Bopp, T. T. T. Chau, Ö. Gürses, T. Ilyina, P. Landschützer, A. Lenton, L. Resplandy, C. Rödenbeck, J. Schwinger, R. Séférian, Consistency and challenges in the ocean carbon sink estimate for the global carbon budget. *Front. Mar. Sci.* **7**, 571720 (2020).

48. N. Mayot, C. Le Quéré, C. Rödenbeck, R. Bernardello, L. Bopp, L. M. Djeutchouang, M. Gehlen, L. Gregor, N. Gruber, J. Hauck, Y. Iida, T. Ilyina, R. F. Keeling, P. Landschützer, A. C. Manning, L. Patara, L. Resplandy, J. Schwinger, R. Séférian, A. J. Watson, R. M. Wright, J. Zeng, Climate-driven variability of the Southern Ocean CO_2 sink. *Philos. Trans. R. Soc. A* **381**, 20220055 (2023).

49. R. Wanninkhof, Relationship between wind speed and gas exchange over the ocean. *J. Geophys. Res. Oceans* **97**, 7373–7382 (1992).

50. R. Arruda, D. Atamanchuk, M. Cronin, T. Steinhoff, D. Wallace, At-sea intercomparison of three underway pCO_2 systems: Intercomparison of pCO_2 systems. *Limnol. Oceanogr. Methods* **18**, 63–76 (2020).

51. L. Olivier, J. Boutin, G. Reverdin, N. Lefèvre, P. Landschützer, S. Speich, J. Karstensen, M. Labaste, C. Noisel, M. Ritschel, T. Steinhoff, R. Wanninkhof, Wintertime process study of the North Brazil Current rings reveals the region as a larger sink for CO_2 than expected. *Biogeosciences* **19**, 2969–2988 (2022).

52. T. H. Heimdal, G. A. McKinley, The importance of adding unbiased Argo observations to the ocean carbon observing system. *Sci. Rep.* **14**, 19763 (2024).

53. A. J. Watson, U. Schuster, J. D. Shutler, T. Holding, I. G. C. Ashton, P. Landschützer, D. K. Woolf, L. Goddijn-Murphy, Revised estimates of ocean-atmosphere CO_2 flux are consistent with ocean carbon inventory. *Nat. Commun.* **11**, 4422 (2020).

54. A. R. Fay, T. H. Heimdal, V. Acquaviva, A. P. Shaum, G. A. McKinley, Sensitivity of ocean carbon sink estimates to rare observations. *Geophys. Res. Lett.* **52**, e2025GL117961 (2025).

55. D. Hohensee, "Vergleich von pCO_2 Sensoren für die Oberfläche des Ozeans - Eine Wirtschaftlichkeitsuntersuchung," thesis, Christian-Albrechts-Universität zu Kiel, Germany (2017).

56. B. R. Carter, N. L. Williams, W. Evans, A. J. Fassbender, L. Barbero, C. Hauri, R. A. Feely, A. J. Sutton, Time of detection as a metric for prioritizing between climate observation quality, frequency, and duration. *Geophys. Res. Lett.* **46**, 3853–3861 (2019).

57. A. R. Gray, The four-dimensional carbon cycle of the Southern Ocean. *Ann. Rev. Mar. Sci.* **16**, 163–190 (2024).

58. A. E. R. Hassoun, T. Tanhua, I. Lips, E. Heslop, G. Petihakis, J. Karstensen, The European ocean observing community: Urgent gaps and recommendations to implement during the UN ocean decade. *Front. Mar. Sci.* **11**, 1394984 (2024).

59. C. Meinig, E. F. Burger, N. Cohen, E. D. Cokelet, M. F. Cronin, J. N. Cross, S. de Halleux, R. Jenkins, A. T. Jessup, C. W. Mordy, N. Lawrence-Slavas, A. J. Sutton, D. Zhang, C. Zhang, Public–private partnerships to advance regional ocean-observing capabilities: A saildrone and NOAA-PMEL case study and future considerations to expand to global scale observing. *Front. Mar. Sci.* **6**, 448 (2019).

60. D. Roemmich, M. H. Alford, H. Claustre, K. Johnson, B. King, J. Moum, P. Oke, W. B. Owens, S. Pouliquen, S. Purkey, M. Scanderbeg, T. Suga, S. Wijffels, N. Zilberman, D. Bakker, M. Baringer, M. Belbeoch, H. C. Bittig, E. Boss, P. Calil, F. Carse, T. Carval, F. Chai, D. Ó. Conchubhair, F. d'Ortenzio, G. Dall'Olmo, D. Desbruyeres, K. Fennel, I. Fer, R. Ferrari, G. Forget, H. Freeland, T. Fujiki, M. Gehlen, B. Greenan, R. Hallberg, T. Hibiya, S. Hosoda, S.

Jayne, M. Jochum, G. C. Johnson, K. Kang, N. Kolodziejczyk, A. Körtzinger, P.-Y. L. Traon, Y.-D. Lenn, G. Maze, K. A. Mork, T. Morris, T. Nagai, J. Nash, A. N. Garabato, A. Olsen, R. R. Pattabhi, S. Prakash, S. Riser, C. Schmechtig, C. Schmid, E. Shroyer, A. Sterl, P. Sutton, L. Talley, T. Tanhua, V. Thierry, S. Thomalla, J. Toole, A. Troisi, T. W. Trull, J. Turton, P. J. Velez-Belchi, W. Walczowski, H. Wang, R. Wanninkhof, A. F. Waterhouse, S. Waterman, A. Watson, C. Wilson, A. P. S. Wong, J. Xu, I. Yasuda, On the future of Argo: A global, full-depth, multi-disciplinary array. *Front. Mar. Sci.* **6**, 439 (2019).

61. C. Whitt, J. Pearlman, B. Polagye, F. Caimi, F. Muller-Karger, A. Copping, H. Spence, S. Madhusudhana, W. Kirkwood, L. Grosjean, B. M. Fiaz, S. Singh, S. Singh, D. Manalang, A. S. Gupta, A. Maguer, J. J. H. Buck, A. Marouchos, M. A. Atmanand, R. Venkatesan, V. Narayanaswamy, P. Testor, E. Douglas, S. de Halleux, S. J. Khalsa, Future vision for autonomous ocean observations. *Front. Mar. Sci.* **7**, 697 (2020).

62. N. P. Mongwe, M. Vichi, P. M. S. Monteiro, The seasonal cycle of pCO_2 and CO_2 fluxes in the Southern Ocean: Diagnosing anomalies in CMIP5 Earth system models. *Biogeosciences* **15**, 2851–2872 (2018).

63. Y. Silvy, T. L. Frölicher, J. Terhaar, F. Joos, F. A. Burger, F. Lacroix, M. Allen, R. Bernadello, L. Bopp, V. Brovkin, J. R. Buzan, P. Cadule, M. Dix, J. Dunne, P. Friedlingstein, G. Georgievski, T. Hajima, S. Jenkins, M. Kawamiya, N. Y. Kiang, V. Lapin, D. Lee, P. Lerner, N. Mengis, E. A. Monteiro, D. Paynter, G. P. Peters, A. Romanou, J. Schwinger, S. Sparrow, E. Stofferahn, J. Tjiputra, E. Tourigny, T. Ziehn, AERA-MIP: Emission pathways, remaining budgets and carbon cycle dynamics compatible with 1.5°C and 2°C global warming stabilization. *Earth Syst. Dynam.* **15**, 1591–1628 (2024).

64. K. D. Six, E. Maier-Reimer, Effects of plankton dynamics on seasonal carbon fluxes in an ocean general circulation model. *Global Biogeochem. Cycles* **10**, 559–583 (1996).

65. C. Heinze, E. Maier-Reimer, A. M. E. Winguth, D. Archer, A global oceanic sediment model for long-term climate studies. *Global Biogeochem. Cycles* **13**, 221–250 (1999).

66. H. Li, T. Ilyina, T. Loughran, A. Spring, J. Pongratz, Reconstructions and predictions of the global carbon budget with an emission-driven Earth system model. *Earth Syst. Dynam.* **14**, 101–119 (2023).

67. B. Liu, K. D. Six, T. Ilyina, Incorporating the stable carbon isotope ^{13}C in the ocean biogeochemical component of the Max Planck Institute Earth System model. *Biogeosciences* **18**, 4389–4429 (2021).

68. J. Maerz, K. D. Six, I. Stemmler, S. Ahmerkamp, T. Ilyina, Microstructure and composition of marine aggregates as co-determinants for vertical particulate organic carbon transfer in the global ocean. *Biogeosciences* **17**, 1765–1803 (2020).

69. W. A. Müller, J. H. Jungclaus, T. Mauritsen, J. Baehr, M. Bittner, R. Budich, F. Bunzel, M. Esch, R. Ghosh, H. Haak, T. Ilyina, T. Kleine, L. Kornblueh, H. Li, K. Modali, D. Notz, H. Pohlmann, E. Roeckner, I. Stemmler, F. Tian, J. Marotzke, A higher-resolution version of the Max Planck Institute Earth System model (MPI-ESM1.2-HR). *J. Adv. Model. Earth Syst.* **10**, 1383–1413 (2018).

70. D. M. Nielsen, F. Chegini, J. Maerz, S. Brune, M. Mathis, M. Dobrynin, J. Baehr, V. Brovkin, T. Ilyina, Reduced arctic ocean CO_2 uptake due to coastal permafrost erosion. *Nat. Clim. Chang.* **14**, 968–975 (2024).

71. A. Jersild, SOMFFN, Version 2022 (2023); https://github.com/aljersild/SOMFFNv2022.

72. B. Nababan, D. Ulfah, J. P. Panjaitan, Light propagation, coefficient attenuation, and the depth of one optical depth in different water types. *IOP Conf. Ser. Earth Environ. Sci.* **944**, 012047 (2021).

73. Global Carbon Project, Supplemental Data of Global Carbon Budget 2022, Version 1.0 (2022); https://doi.org/10.18160/gcp-2022.

74. T. Naegler, Reconciliation of excess ^{14}C-constrained global CO_2 piston velocity estimates. *Tellus B* **61**, 372–384 (2022).

75. P. J. Duke, R. C. Hamme, D. Ianson, P. Landschützer, N. C. Swart, P. A. Covert, High-resolution neural network demonstrates strong CO_2 source-sink juxtaposition in the coastal zone. *J. Geophys. Res. Oceans* **129**, e2024JC021134 (2024).

76. Y.-H. Park, T. Park, T.-W. Kim, S.-H. Lee, C.-S. Hong, J.-H. Lee, M.-H. Rio, M.-I. Pujol, M. Ballarotta, I. Durand, C. Provost, Observations of the antarctic circumpolar current over the udintsev fracture zone, the narrowest choke point in the Southern Ocean. *J. Geophys. Res. Oceans* **124**, 4511–4528 (2019).
